# Electroconvulsive therapy during pregnancy: a systematic review of case studies

**DOI:** 10.1007/s00737-013-0389-0

**Published:** 2013-11-24

**Authors:** Kari Ann Leiknes, Mary Jennifer Cooke, Lindy Jarosch-von Schweder, Ingrid Harboe, Bjørg Høie

**Affiliations:** 1 Norwegian Knowledge Centre for the Health Services, Box 7004 St. Olavsplass, Pilestredet Park 7, Oslo, 0130 Norway; 2 Department for Psychosis, Psychiatric Clinic, Haukeland University Hospital, Bergen, 5021 Norway; 3 Division of Psychiatry, Tiller DPS and Faculty of Medicine, Institute of Neuroscience, St. Olav’s University Hospital and Norwegian University of Science and Technology (NTNU), P O Box 3008, Lade, 7441 Trondheim, Norway

**Keywords:** Electroconvulsive therapy, Pregnancy, Mental disorders, Review, Systematic

## Abstract

This study aims to explore practice, use, and risk of electroconvulsive therapy (ECT) in pregnancy. A systematic search was undertaken in the databases Medline, Embase, PsycINFO, SveMed and CINAHL (EBSCO). Only primary data-based studies reporting ECT undertaken during pregnancy were included. Two reviewers independently checked study titles and abstracts according to inclusion criteria and extracted detailed use, practice, and adverse effects data from full text retrieved articles. Studies and extracted data were sorted according to before and after year 1970, due to changes in ECT administration over time. A total of 67 case reports were included and studies from all continents represented. Altogether, 169 pregnant women were identified, treated during pregnancy with a mean number of 9.4 ECTs, at mean age of 29 years. Most women received ECT during the 2nd trimester and many were Para I. Main diagnostic indication in years 1970 to 2013 was Depression/Bipolar disorder (including psychotic depression). Missing data on fetus/child was 12 %. ECT parameter report was often sparse. Both bilateral and unilateral electrode placement was used and thiopental was the main anesthetic agent. Adverse events such as fetal heart rate reduction, uterine contractions, and premature labor (born between 29 and 37 gestation weeks) were reported for nearly one third (29 %). The overall child mortality rate was 7.1 %. Lethal outcomes for the fetus and/or baby had diverse associations. ECT during pregnancy is advised considered only as last resort treatment under very stringent diagnostic and clinical indications. Updated international guidelines are urgently needed.

## Introduction

For patients with severe psychiatric disorders in the pregnancy period, either medication resistant illness, extremely high suicide risk, psychotic agitation, severe physical decline due to malnutrition or dehydration, electroconvulsive therapy (ECT) still appears as a strong option (Berle et al. 2011; 2003). Previous review publications have advocated ECT to be a relatively safe during pregnancy (Anderson and Reti 2009; Miller 1994; Reyes et al. 2011; Saatcioglu and Tomruk 2011). International ECT guidelines have no clear statements about pregnancy being a contraindication (American Psychiatric 2001; Enns et al. 2010; Royal College of Psychiatrists 2005). Checklists for when ECT is an option during pregnancy have also been provided in textbooks of interface between gynecology and psychiatry (Stewart and Erlick Robinson 2001), without mention of any potential risks to be taken into account.

Prevalence of major depressive episode (MME) during pregnancy is estimated at 12.4 % (Le et al. 2011). Considering that depression is the most common mental disorder (63 %), followed by bipolar disorder (43 %) and schizophrenia (13 %) among deliveries to women with atypical antipsychotic use (Toh et al. 2013), the decision of ECT during pregnancy would not appear uncommon. Although prevalence data on ECT administered during pregnancy is not retrievable, and ECT clearly rarely used during pregnancy in most clinical settings as illustrated by a recent review of contemporary use and practice of ECT worldwide (Leiknes et al. 2012), ECT was noted administered during pregnancy at 10 Polish sites (Gazdag et al. 2009) and also in Spain (Bertolin-Guillen et al. 2006).

Administration of psychotropic drugs during pregnancy requires great caution and benefits must be weighed against potential risks, especially in the first trimester (Stewart and Erlick Robinson 2001). Although evidence for psychotropic medication teratogenicity is generally lacking or limited (Gentile 2010), mood stabilizers such as lithium and valproate are strongly discouraged (Berle and Spigset 2003; Gentile 2010) and carbamazapine controversial (Gentile 2010; Stewart and Erlick Robinson 2001). As for antidepressants, a recent population-based cohort study data from the Danish Fertility Database has found no associated risk with use of SSRIs during pregnancy (Jimenez-Solem et al. 2013). For antipsychotics the risk associated with use during pregnancy is unclear (McCauley-Elsom et al. 2010).

In a systematic review concerning children of women with epilepsy (WWE), no support was found for the common view that epilepsy per se represented a risk for increased congenital malformations (Fried et al. 2004). Conversely, a large population-based register study found a twofold overall risk of malformation in the offspring from WWE compared with those without epilepsy (Artama et al. 2006). Caesarian section in WWE has, also been found to be performed twice as frequently compared with the general population (Olafsson et al. 1998). Total prevalence of major congenital anomalies, is by a large European study (Dolk et al. 2010) reported as 23.9 per 1,000 births for 2003–2007 and 80 % live births. Prevalence of congenital heart disease (the most common birth defect) to be 4–6/1,000 live births by another USA study (Ermis and Morales 2011).

In a previous review of the literature from 1941 to 2007 undertaken by Anderson and Reti (2009), with 57 included studies, ECT was reported administered to 339 women during pregnancy. The same review also reports a partial positive ECT response for pregnant women together with a very low number (*N*= 11) of ECT-related fetal or neonatal abnormalities. Whether these numbers can be reaffirmed and whether there is enough support for APAs the statement that ECT treatment has a “low risk and high efficacy in the management of specific disorders in all three trimesters of pregnancy” (American Psychiatric 2001) is a concern for this present review.

Treatment of mental disorders in pregnancy poses a unique clinical challenge due to potential effects also on the fetus from the intervention. As ECT is utilized worldwide and predominantly in the treatment of women (Leiknes et al. 2012), updated knowledge about safety and risk of ECT treatment during pregnancy for both the mother and fetus/child is of utmost primary importance.

Against this background, the main objective of this article is to give a systematic case overview of ECT administered during pregnancy, with newer date studies in mind, as well as to report the potential harm (adverse events for mother and fetus/baby).

## Materials and methods

### Data sources and search strategy

A systematic literature search was undertaken in the following databases: Ovid MEDLINE, Embase (Ovid) PsycINFO (Ovid), SveMed, Ovid Nursing Database and CINAHL (EBSCO) (Table 5 in Appendix 1) in September 2010. The search was updated in January and November 2012 and supplemented with ISI web of Knowledge, Clinical Trials.gov, PROSPERO (CRD), WHO ICTRP, POP-database (Table 6 in Appendix 1). Search terms intended for Medline were adapted (such) as required for the other databases. Subject headings and free text words used were “electroconvulsive therapy,” “electroshock,” “electroconvulsive,” “ECT,” combined with “pregnancy” or “pregnant women” and any of the following “antenatal,” “prenatal,” “perinatal,” “gravid,” or “gestation” limited to human studies and dating until today. The search did not exclude the postpartum period to make sure that no articles on the topic were missed. No date limitation was set to find all possible earliest published cases from the 1940s. Relevant references, known to authors of this review from earlier published reviews on this topic or reference lists in retrieved included papers, were also found by hand.

### Inclusion and exclusion criteria

#### Inclusion criteria

Studies in the following languages were included: English, Norwegian, Swedish, Danish, Dutch, French, Italian, and Spanish. In addition to authors’ European language fluency, the online Google translation tool (http://translate.google.com/) was used when needed.

#### Exclusion criteria

Exclusion criteria include not a data-based study, no or unclear report of ECT undertaken during pregnancy, pseudocyesis, ECT undertaken only in the postpartum period, and not during pregnancy.

### Screening of literature

Two reviewers (Kari Ann Leiknes (KAL) and Bjørg Høie (BH)) independently checked the titles, and where available, the abstracts of the studies identified by the electronic database searches. All references appearing to meet inclusion criteria, including those with insufficient details were requested in full text. Reviewers (KAL, BH, and Mary J. Cooke (MJC)), consisting of two pairs independently extracted data from the retrieved full-text articles according to a pre-designed data extraction scheme. All discrepancies were resolved by consensus meeting/discussion, and the final decision was made by the first author (KAL). Ingrid Harboe (IH) undertook the extensive updated literature search. All authors (including Lindy Jarosch-von Schweder (LJS) have contributed to the data presentation and manuscript text.

### Data extraction

Briefly, the following aspects were considered: ECT practice and use; publication year and country; diagnoses/indication; mother’s age; number of pregnancies (primipara (P1), multipara (P2, 3), etc.); time ECT was administered according to number of gestation weeks (GW), 1st trimester (≤13 GW), 2nd trimester (14–26 GW), 3rd trimester (≥27 GW); total number ECTs administered, ECT administration frequency (two to three times week); ECT parameters (i.e., the manner in which ECT is applied: brief pulse or sine wave current, device type, electrode placement bilateral (BL) or unilateral (UL)); anesthesia type and monitoring (of both mother and fetus); time of birth; and adverse events mother (e.g., genital bleeding, miscarriage, eclampsia, and still birth) and/or baby (e.g., fetal malformations, Apgar score, etc.). As ECT treatment has changed over the years, as for use of anesthesia (termed modified ECT as opposed to unmodified ECT, without anesthesia), device and type of current (mainly from sine wave to brief pulse wave), a clinical cut off for presenting the extracted data was set at 1970.

## Results

### Study selection

**Fig. 1:**
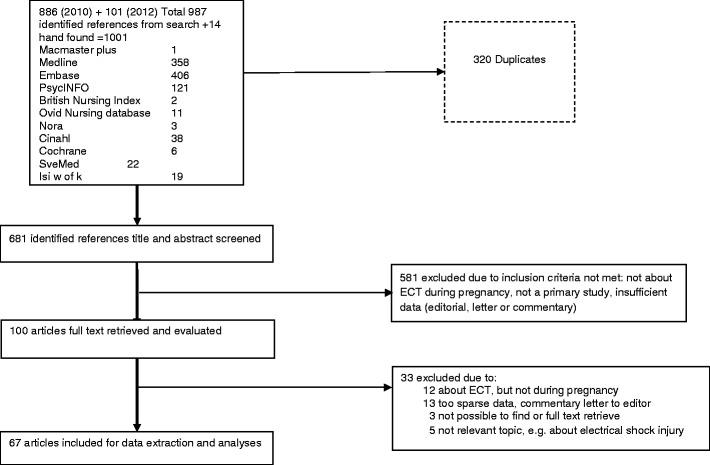
Flow chart of the study selection process

The study selection process, databases searched, and references identified are given in Fig. 1. Altogether, 1,001 references were identified: 681 titles and abstracts screened, 100 full texts screened, 67 included for data extraction, and 33 full texts excluded.

### Description of studies

Overview of included case studies (*N* = 67) according to descending publication year, country represented, number of pregnancy cases and fetus and/or baby cases reported are given in Table 1. Overview of full text excluded studies (*N* = 33) and reasons for exclusion are given in Appendix 2. Twelve references were found not relevant to topic (about ECT, but not in pregnancy, e.g., in postpartum or other conditions), 13 had insufficient/too sparse data, 3 were impossible to find/full text retrieve, and 5 were not relevant, for example, only about anesthesia types or electrical shock accident injury during pregnancy. Detailed extracted data from each included study, such as diagnostic indication, ECT parameters, report of effect and events are presented in Summary of findings tables (*N* = 67), Appendix 3.

**Table 1:** Overview of included studies (*N* = 67), publication year, country, number of pregnancy, and fetus/baby cases

Primary Author and Year	Country	Number of pregnancy cases	Number of fetus (F) or baby (B) cases
De Asis et al. (2013)	USA	1	1
Gahr et al. (2012)	Germany	1	1 F
Yang et al. (2011)	South Korea	1	1
O’Reardon et al. (2011)	USA	1	1
Salzbrenner et al. (2011)	USA	1	1
Lovas et al. (2011)	Hungary	1	1
Pesiridou et al. (2010)	USA	1	1
Serim et al. (2010)	Turkey	1	1
Molina et al. (2010)	Spain	2	2
Kucukgoncu et al. (2009)	Turkey	1	1
Ghanizadeh et al. (2009)	Iran	1	1 F
Malhotra et al. (2008)	India	2	–
Ceccaldi et al. (2008)	France	1	1
Bozkurt et al. (2007)	Turkey	1	1
Kasar et al. (2007)	Turkey	1	1
Pinette et al. (2007)	USA	1	1
Espínola-Nadurille et al. (2007)	Mexico	1	1 F
Prieto Martin et al. (2006)	Spain	1	1
Balki et al. (2006)	Canada	1	1 F death
Maletzky (2004)	USA	4	1 (3 unknown)
Brown et al. (2003)	USA	1	–
DeBattista et al. (2003)	USA	1	1
Fukuchi et al. (2003)^a^	Japan (Japanese)	1	–
Ishikawa et al. (2001)^a^	Japan (Japanese)	1	1 F
Iwasaki et al. (2002)	Canada	1	1
Polster and Wisner (1999)	USA	1	–
Gilot et al. (1999)	France	1	1 B death
Bhatia et al. (1999)	USA	2	2
Echevarria et al. (1998)	Spain	1	1 F death
Livingston et al. (1994)	USA	1	1 (twins)
			1 B death
Verwiel et al. (1994)	Netherlands	1	1
Vanelle et al. (1991)	France	5	4
			1 F death
Sherer et al. (1991)	USA	1	1
Yellowlees and Page (1990)	Australia	1	1
LaGrone (1990)	USA	1	1
Griffiths et al. (1989)	USA	1	1
Mynors-Wallis (1989)	UK	1	–
Varan et al. (1985)	Canada	1	1
Dorn (1985)	USA	1	–
Wise et al. (1984)	USA	1	–
Repke and Berger (1984)	USA	1	1
Loke and Salleh (1983)	Malaysia	3	3
Impastato et al. (1964)	USA	1	1
Evrard (1961)	Belgium	1	1
Barten (1961)	Netherlands	2	2
Ferrari (1960)	Italy	8	7
			1 B death
Sobel (1960)	USA	33	31
			2 B deaths
Schachter (1960)	France	1	1
Smith (1956)	UK	15	15
Monod (1955)	France	4	3
Laird (1955)	USA	8	8
Russell and Page (1955)	UK	10	–
Charatan and Oldham (1954)	UK	1	1
Wickes (1954)	UK	1	1
Yamamoto et al. (1953)	USA	1	1
Forman et al. (1952)	USA	2	2
Cooper (1952)	South Africa	1	1
Porot (1949)	Alger	3	3
Plenter (1948)	Dutch	3	2
			1 F death
Simon (1948)	USA	3	2
			1 B death
Doan and Huston (1948)	USA	7	7
Boyd and Brown (1948)	USA	2	1
Block (1948)	New York, USA	1	1
Kent (1947)	New York, USA	3	2
			1 F death
Gralnick (1946)	New York, USA	1	1 F death
Polatin and Hoch (1945)	New York, USA	2	–
Thorpe (1942)	UK	1	1

^a^Japanese language, English abstract

A total of 67 case report studies were included, 42 (63 %) from 1970 to 2013 and 25 (37 %) from 1942 to 1970 (Table 1). The literature search included all years, but no studies according to inclusion criteria of this review were found in the 1970s (see Appendix 2 for two excluded 1970s studies (Levine and Frost 1975; Remick and Maurice 1978) lacking ECT data). Studies from all continents were represented as follows: North America (USA and Canada), 32; South America, 1; Europe, 25; Asia (including Middle East), 6; Africa, 2; and Australia, 1. A total of 169 pregnant women were ECT treated from 1942 to 2013. Reports on the fetus or newborn baby/child were found for only 148 cases resulting in 12 % “missing” fetus/baby data (see Table 1).

Altogether, 169 ECT treated pregnant women were identified, exposed to a total number of 1,187 ECTs. Mean and standard deviation (M (SD)) number of ECTs administered per pregnant woman was 9.4 (6.4). Mean age (M (SD) in years) of pregnant women treated with ECT was 28.9 (6.2) and age range 16½–48 years. Overview of ECT-treated pregnant women, number of ECTs, and diagnoses, after and before 1970 is given in Table 2.

**Table 2:** ECT-treated pregnant women, number of ECTs, and diagnoses before and after 1970

	Years 1970 to 2013	Years 1942 to 1970	All years
Number of ECT treated pregnant women (*N*)	54	115	169
Age in years (M (SD))	28.8 (6.0)	28.9 (6.4)	28.9 (6.2)
Total number of ECTs administered	446	741	1,187
Number of ECTs administered (M (SD))	8.5 (4.2)	10.2 (7.2)	9.4 (6.4)
Diagnoses in percent (%)			
Depression, bipolar	63	35	43
Schizophrenia, psychosis	28	50	43
Other (anxiety, obsessive–compulsive disorder, etc.)	9	4	6
(Missing diagnoses)	(−)	(11)	(8)
Percent (%) Para1 within number of women	39 %	17 %	24 %
Number of fetus and/or baby reported	47	101	148
Number and percent (%) missing within	7 (13 %)	14 (12 %)	21 (12 %)

Almost two thirds (63 %) diagnostic indication for ECT was Depression/Bipolar disorder (including psychotic depression) from year 1970 until today (2013), but Schizophrenia and other diagnoses the main indication (54 %) from 1942 until 1970 (Table 2). Diagnostic data was not missing in any reports from 1970 to 2013, but missing (15 %) and sometimes very unclear in several earlier reports from 1942 to 1970. Category of “other” diagnoses included obsessive–compulsive disorder (OCD) (Barten 1961; Fukuchi et al. 2003), generalized anxiety with panic attacks (Bhatia et al. 1999; Simon 1948), and Neuroleptic Malignant Syndrome (NMS) (Verwiel et al. 1994).

Altogether 21 out of 54 (39 %) women were nullipara (Para1) in the later years (from 1970 to 2013) (Table 2) and for one case in 2011 the pregnancy was by in vitro fertilization (Salzbrenner et al. 2011). The latest ECT administered in pregnancy was at 40 GW (Laird 1955; Schachter 1960) and the earliest at 4 GW (1955). Information about which pregnancy trimester the ECT was undertaken or started was found for 121 women out of 169 (28 % missing). Overview of the ECT reports according to pregnancy trimester for these 121 women is given in Table 3. Most women (53 %) received ECT during the 2nd trimester, although use in the 1st trimester was not uncommon (16 %) and for some, ECT was conducted throughout the entire pregnancy (Pinette et al. 2007).

**Table 3:** ECT-treated women (*N* = 121) by pregnancy trimesters

	1st trimester (≤13 GW)	2nd trimester (14–26 GW)	3rd trimester (≥27 GW)
Number of women (*N* (%))	19 (16 %)	64 (53 %)	38 (31 %)
Age in years (M (SD))	29.3 (5.1)	28.3 (5.9)	28.4 (6.8)
Number of ECTs (M (SD)) administered	10.7 (6.4)	11.1 (7.5)	7.1(3.1)
Para percent (%)			
Primipara (P1)	37 (P1)	36 (P1)	32 (P1)
Multipara (≥P2)	42 (≥P2)	37 (≥P2)	47 (≥P2)
(Missing)	(21)	(27)	(21)
Diagnoses (%)			
Depression, bipolar	63	66	63
Schizophrenia, psychosis	32	28	30
Other	5	5	3
(Missing)	(0)	(1)	(4)

Generally, the data reported in all studies was very varied concerning the ECT intervention per se, the setting of administration, monitoring, and outcome for both mother and fetus/child.

### ECT practice during pregnancy

The setting in which the ECT was administered was usually not recorded. However, ECT undertaken in a surgical-obstetric recovery room or delivery environment was noted by three (Gilot et al. 1999; Wise et al. 1984; Yellowlees and Page 1990).

Monitoring of mother before, during, and after varied. In addition, monitoring of fetus varied greatly from some monitoring to no fetal monitoring by Vanelle et al. (1991). There was some use of cardiotocography (Molina et al. 2010; O’Reardon et al. 2011; Verwiel et al. 1994) but cardiotocography was also noted as not being useful in early pregnancy (before 24 GW) by Lovas et al. (2011). Mother in tilt position during ECT was used in some reports (Brown et al. 2003; Gilot et al. 1999; Livingston et al. 1994; Malhotra et al. 2008; Yang et al. 2011) and by others tilt position was reported not used (Bhatia et al. 1999; Bozkurt et al. 2007; DeBattista et al. 2003).

ECT parameters, such as electrical current type (brief pulse or sine wave), placement of electrodes (UL, BL, bitemporal, and bifrontal) and device manufacture type used was noted in most studies of later date but otherwise very sparsely. (See summary of findings table, Appendix 3). UL placement of electrodes was noted in six studies (Balki et al. 2006; Gahr et al. 2012; Pesiridou et al. 2010; Varan et al. 1985; Wise et al. 1984; Yellowlees and Page 1990).

Data on anesthetic agents used combined with muscle relaxant, premedication and 100 % oxygenation was mainly stated in the later date studies (1970 to 2013). Although 13 % of these later date studies (1970 to 2013) were missing anesthesia data, a trend was seen for the following being most used: thiopental (22 %), methohexital (15 %), and propofol (17 %). Anesthesia induced reduced fetal heart rate (FHR) was noted with propofol but not thiamylal in an ECT pregnancy case by Iwasaki et al. (2002). In addition, severe fetal bradycardia by methohexital but not with following propofol anesthesia during ECT administration by De Asis et al. (2013). To avoid pulmonary aspiration, tracheal intubation was preferred by Malhotra et al. (2008) when pregnancy was beyond 1st trimester.

Unmodified (without anesthesia) ECT was noted in the earlier studies (from 1942 to 1970), such as in all 8 cases reported by Laird (1955) and in 6 out of 15 cases by Smith (1956). Even use of only muscle relaxant without anesthesia was noted in 7 ECT pregnancy cases by Doan and Huston (1948).

### Fetus, baby/child—monitoring, and follow-up

Fetus or baby/child data was sometimes totally absent even in the later date studies, such as in Gahr et al. (2012) and Ghanizadeh et al. (2009) as well as some earlier ones, for example Russell and Page (1955). Some reported new born baby Apgar score and weight, but most often the information on the newborn infant was meager and the condition of baby/child noted as normal, “healthy baby,” or nothing abnormal.

Information about monitoring of fetus during ECT varied greatly from none at all, to obstetric consultations and ultrasonography between treatment sessions (Espínola-Nadurille et al. 2007; Kasar et al. 2007; Serim et al. 2010) to before and after FHR and Doppler monitoring (O’Reardon et al. 2011).

Although most studies had no follow-up data on the children, some had sparsely noted follow-up at 1 month (Repke and Berger 1984), 3 months (Yellowlees and Page 1990), 18 months (O’Reardon et al. 2011), 2 weeks to 5 months (Sobel 1960), 2½ years (Yamamoto et al. 1953), and 6 years (Evrard 1961). A more detailed follow-up study from 1955 by Forssman (1955) of 16 children, whose mothers were given ECT during pregnancy between years 1947 and 1952, was excluded since it contained only data on the children without any ECT during pregnancy data on the mothers.

### ECT risk and adverse events

No deaths of mother/ECT treated pregnant patient were found in any studies. Overall (all years), child mortality rate was 7.1 % (12/169), and from 1970 to 2013 mortality rate was 9.4 % (5/54) and from 1942 to 1970, 6.1 % (7/115) (see Table 1). Lethal outcomes for the fetus and/or baby were stated to have diverse causes, in one case a long lasting severe grand mal seizure (status epilepticus) induced by ECT (Balki et al. 2006). A combination of insulin coma treatment and ECT was found for 3 early studies in the period 1946 to 1954 by Kent (1947), Gralnick (1946), Wickes (1954)—all with severe very adverse outcome for the fetus/baby. Overview of all reported adverse events for ECT treated pregnant women and fetus and/or baby child are given in Table 4.

**Table 4:** Overview of reported adverse events for ECT-treated pregnant women and fetus and/or baby found in all included (*N* = 67) studies

	Year period of events			Studies by first author with event reported according to trimester			Comments
	Years 1970 to 2013	Years 1942 to 1970	All years	1st (unknown)	2nd	3rd	
Event type mother (*n* (%))							
Vaginal bleeding	3 (7 %)	5 (23 %)	8 (12 %)	Ghanizadeh et al. (2009), Echevarria et al. (1998), and Ferrari (1960)^a^	Sherer et al. (1991) and Boyd and Brown (1948)^a^	Porot (1949) ^a^	2 events in Porot (1949) and 2 events in Boyd and Brown (1948); vaginal bleeding after each ECT session in Ghanizadeh et al. (2009) and in 1 case Ferrari (1960); abruptio placentae in Sherer et al. (1991)
Uterine contractions	14 (30 %)	2 (9 %)	16 (24 %)	Fukuchi et al. (2003)	Ceccaldi et al. (2008), Polster and Wisner (1999), Sherer et al. (1991), Ishikawa et al. (2001), and Boyd and Brown (1948)^a^	Pesiridou et al. (2010), Yang et al. (2011), Serim et al. (2010), Molina et al. (2010), Kasar et al. (2007), Prieto Martin et al. (2006), and Bhatia et al. (1999)	2 events in Bhatia et al. (1999), Boyd and Brown (1948), and Molina et al. (2010)
Abdominal pain	2 (4 %)	4 (18 %)	6 (9 %)	Lovas et al. (2011) and Bozkurt et al. (2007)	Impastato et al. (1964)^a^ and Plenter (1948)^a^	Sobel (1960)^a^	2 events in Sobel (1960)
Miscarriage	3 (7 %)	2 (9 %)	5 (7 %)	Vanelle et al. (1991) Echevarria et al. (1998)	Balki et al. (2006), Plenter (1948),^a^ and Kent (1947)^a^		1 event in Kent (1947)^a^ with also insulin coma treatment
Preeclampsia	2 (4 %)	–	2 (3 %)	Lovas et al. (2011)	Pinette et al. (2007)		
Premature labor (born between 29–37 GW)	13 (28 %)	6 (27 %)	19 (28 %)	Schachter (1960),^a^ Laird (1955),^a^ and Doan and Huston (1948)^a^	Ceccaldi et al. (2008) Gilot et al. (1999), Livingston et al. (1994), LaGrone (1990), and Boyd and Brown (1948)^a^	Pesiridou et al. (2010), Yang et al. (2011), Kasar et al. (2007), Pinette et al. (2007), Prieto Martin et al. (2006), Bhatia et al. (1999), Sherer et al. (1991), Yellowlees and Page (1990), and Wise et al. (1984)	3 events in Doan and Huston (1948) ^a^
Caesarian section births	9 (20 %)	3 (14 %)	12 (17 %)	Lovas et al. (2011)	O’Reardon et al. (2011), Gilot et al. (1999), LaGrone (1990), Laird (1955),^a^ Forman et al. (1952),^a^ and Kent (1947)^a^	Yang et al. (2011), Salzbrenner et al. (2011), Serim et al. (2010), Kasar et al. (2007), and Sherer et al. (1991)	6 born between 29–37 GW; emergency caesarian in Yang et al. (2011) and 1 event in Kent (1947) also insulin coma treatment
Total number of events (*N*)	46	22	68				
Events ratio per number of ECT treated pregnant women within group	0.85 (46/54)	0.19 (22/115)	0.40 (68/169)				
Events ratio (excluding Caesarian section) per number of ECT treated pregnant women within group	0.69 (37/54)	0.16 (19/115)	0.33 (56/169)				
Event type fetus/baby child, number, and percent (*n* (%))							
Fetal cardiac arrhythmias, bradycardia (reduced fetal heart rate (FHR))	13 (54 %)	2 (18 %)	15 (43 %)	Bozkurt et al. (2007) and Dorn (1985)	DeBattista et al. (2003), Iwasaki et al. (2002), Gilot et al. (1999), and Livingston et al. (1994)	De Asis et al. (2013), Serim et al. (2010), Molina et al. (2010), Ishikawa et al. (2001), Prieto Martin et al. (2006), Bhatia et al. (1999), Sherer et al. (1991), and Barten (1961)^a^	Severe reduced FHR with methohexital but not with propofol anesthesia in De Asis et al. (2013), 2 events in Molina et al. (2010), reduced FHR with propofol but not with thiamylal anesthesia in Iwasaki et al. (2002), and 2 events in Barten (1961)^a^
Meconium-stained amniotic fluid	–	1 (9 %)	1 (3 %)			Barten (1961)^a^	
Stillbirth and neonatal death (miscarriage/abortion, fetal death NOT included here)	6 (25 %)	2 (18 %)	8 (23 %)	Gralnick (1946)^a^	Gilot (1999), Livingston et al. (1994), Simon (1948), ^a^ and Kent (1947)^a^	Ferrari (1960)^a^ and Sobel (1960)^a^	2 deaths at full-term. Time baby died after birth: 0 days in Livingston et al. (1994), Gralnick (1946)^a^ and Sobel (1960) ^a^; 2 days in Simon (1948) ^a^; 8 days in Ferrari (1960) ^a^ due to bronchopneumonia; 9 days in Gilot et al. (1999) due to metabolic postsurgical complications after meconium peritonitis treatment in Sobel (1960) ^a^: 1 anencephalic, 1 lung cysts, and bronchopneumonia, died shortly after birth
Neonatal respiratory distress	–		1 (3 %)		LaGrone (1990)		
Bilirubinemi	1 (4 %)	–	1 (3 %)		Verwiel et al. (1994)		
General mental impairment (retarded)	–	2 (18 %)	2 (5 %)		Yamamoto et al. (1953)^a^ and Wickes (1954)^a^		Eye strabismus and mentally impaired (child 2½ years) (Yamamoto et al. 1953).^a^ Blindness and severe mentally retarded (3 years old) (Wickes 1954)^a^ in a case with also insulin coma treatment early in pregnancy
Fetal malformations (teratogenicity)	4 (17 %)	3 (27 %)	7 (20 %)	Schachter (1960)^a^	Livingston et al. (1994) and LaGrone (1990)	Yang et al. (2011), Pinette et al. (2007), and Sobel (1960)^a^	Hyaline membrane disease and congenital hypertrophic pylonic stenosis (Yang et al. 2011); small left cerebellum, bi-hemispheric deep white matter cortical infarct (Pinette et al. 2007); transposition of great vessels, anal atresia, sacral defect, and coarctation of aorta (Livingston et al. 1994); infant growth retardation (LaGrone 1990); severe mental defect, congenital glaucoma, cleft palate (Schachter 1960)^a^; anencephalia (Sobel 1960)^a^; congenital lung cysts (Sobel 1960)^a^
Total number (*N*) events fetus/baby	24	11	35				
Events ratio per number of fetus/baby child within group	0.51 (24/47)	0.11 (11/101)	0.24 (35/148)				

^a^Case studies from 1942 until 1970

Report of adverse advents was high for both pregnant women and fetus/child in studies of later date period (1970 to 2013) compared with earlier date period (1942 to 1970) (see Table 4). Vaginal bleeding was reported more often during the 1st trimester, whereas uterine contractions, premature labour and caesarian sections occurred during 2nd and 3rd trimesters. The use of tocolytic treatment after ECT in order to avoid preterm labor was also noted by several (Fukuchi et al. 2003; Malhotra et al. 2008; Polster and Wisner 1999; Prieto Martin et al. 2006; Serim et al. 2010; Yang et al. 2011), as well as use of prophylactic tocolytic medication before ECT (Malhotra et al. 2008; Polster and Wisner 1999).

## Discussion

### Main findings

Altogether 169 ECT treated pregnant women of mean age 29 years, were identified. They were treated with mean number of ECTs 9.4, as treatment for mainly (62 %) severe “psychotic” depression/bipolar disorder. Half (53 %) of pregnant women received ECT during the 2nd trimester. ECT in the 1st trimester was not uncommon (16 %) and for some, ECT was conducted throughout the entire pregnancy. Altogether, 24 % women were nullipara (Para1). Fetus and/or baby report was found missing for 12 %. Child mortality rate was overall (all years) 7.1 %. A total of 67 adverse events were found among 169 women (rate, 0.40). Most common adverse event for mother was premature labor (born between 29 and 37 GW) 19/67 (28 %) and tocolytic treatment often noted. A total of 35 adverse events were found among the reported 148 fetus/baby children (rate 0.24). The most common reported adverse event for fetus/baby child occurring during the ECT intervention was reduced FHR 15/35 (43 %).

Whether the reduced FHR event is attributable to the ECT intervention per se or to the anesthetic agent or to both is not possible to say from such descriptive case studies. Due to the complexity of the ECT indication, the intervention per se, previous or concomitant psychotropic medication or other complicating somatic or genetic factors, direct causal inference is not possible to take from case studies. This being said though, having in mind that the risk of fetal malformation in WWE is twofold higher (Artama et al. 2006), and caesarian section performed more often among WWE (Olafsson et al. 1998), the potential risk involved with ECT induced epileptogenic seizures must in each case be considered. Such as illustrated in the recent publication by De Asis et al. (2013), where the ECT induced prolonged seizure duration occurred alongside severe reduced FHR and emergency Caesarian section prepared, but later abandoned when the FHR returned to normal. An earlier study (Balki et al. 2006) also reports severe ECT induced status epilepticus with lethal outcome for the fetus/child.

As for the overall occurrence of serious adverse events, such as stillbirth/neonatal death 8/35 (23 %) and fetal malformation 7/35 (20 %), the rates appear higher than that reported in the general population, i.e. 2.3 % major congenital abnormalities and 80 % live births (2010) and 0.6 % congenital heart disease (Ermis and Morales 2011). Some included studies though claim the miscarriage rate not to be higher than in the general population (Malhotra et al. 2008) and ECT to be less risky than pharmacological treatment (Kasar et al. 2007). However, figures from case studies cannot directly be compared with figures from large observational prevalence studies. This being said, close monitoring of mother and fetus during and after ECT treatment taking into regard the trimester situation, is crucial to bear in mind, such as use of cardiotocography, ultrasound between treatments, tilt position for mother including tocolytic treatment to prevent preterm labor. All these monitoring factors varied greatly in the included studies.

Direct effect of anesthetic agents on the fetus is still relatively unknown (Iwasaki et al. 2002). FHR variability and reduction under the ECT intervention is often mentioned as something to expect to happen. Propofol’s known associated risk of bradycardia calls for alertness from a fetal cardiovascular viewpoint and extra caution is needed where the fetus is immature or has cardiovascular complications. Thiopental (22 %), methohexital (15 %), and propofol (17 %) are the most used anesthetic agents. However, case studies with both anesthesia in favor of propofol (De Asis et al. 2013) and that against it (Iwasaki et al. 2002) are published.

Some factors to bear in mind in the different pregnancy trimesters are mentioned below:

1. Knowledge about when and how to administer ECT in early pregnancy, in order to reduce risk for both mother and fetus, is limited. Cardiotocography monitoring for the fetus, in this early period (before 24 GW) is not so feasible (Lovas et al. 2011). Risk of post ECT vaginal bleeding (indicative of abruptio placenta) and abortion (Vanelle et al. 1991) is mentioned. The complexity of any causal attribution to ECT is illustrated in the case by Yang (Yang et al. 2011) reporting congenital hyaline membrane disease and hypertrophic pyloric stenosis in a premature baby delivered by emergency section, since the mother had been treated with an extensive amount of antipsychotic and antidepressant medication prior to admission due to a 15 year long history of schizophrenia.
2. Transient FHR reduction (bradycardia) arising during the ECT and subsiding afterwards is commonly reported from this trimester period, likewise post-ECT uterine contractions. The need for both pre- and post-ECT tocolytic treatment in order to avoid preterm labor is considerable (Fukuchi et al. 2003; Malhotra et al. 2008; Polster and Wisner 1999; Prieto Martin et al. 2006; Serim et al. 2010; Yang et al. 2011).
3. Tilt position is recommended by several, especially in the last trimester in order to reduce risk of gastric reflux. Also inhalation anesthesia is pointed out by Ishikawa et al. (2001) to be beneficial in the last stages of pregnancy in order to reduce uterine contraction and potential uterine relaxation effect of anesthetics.

The overall total number of included studies (*N* = 67) in our review is larger than the 57 by Anderson and Reti (2009). However, overall total number of ECT treated pregnant women (*N* = 169) is much less than the 339 by the same authors (Anderson and Reti 2009). Unlike the Anderson and Reti (2009), numbers of ECT treated pregnant women referred to by others in the general text of the case article, have not been included in this review. Strictly according to the predetermined review criteria, only direct case reports by the study authors are included in the total count number (169) of pregnant ECT treated women by us. For example, only one case is included in this review from the publication by Impastato et al. (1964) as opposed to 159 cases by Anderson and Reti (2009), and we have not included the Forssman (1955) follow-up of 16 infants/children on ECT treated mothers, since this study contains no ECT pregnancy data, i.e. data on the mothers treatment. Likewise the study by Levine and Frost (1975) is excluded by us, since it only contained information about anesthesia type and cardiovascular responses to ECT in a 3rd semester pregnancy and no other information.

Previous studies, such as that by O’Reardon et al. (2011) and previous reviews (Anderson and Reti 2009; Miller 1994; Saatcioglu and Tomruk 2011) as well as international guidelines (American Psychiatric 2001; Enns et al. 2010; Royal College of Psychiatrists 2005) and recent textbooks (Stewart and Erlick Robinson 2001) have regarded ECT to be relatively safe during all trimesters of pregnancy. Contrary to this standpoint, our review and overview of recorded adverse events from all case studies call for great clinical caution. Voices of concern, similar to ours, appear also in the included study Pinette et al. (2007) and APA statements regarding ECT as a safe intervention during pregnancy questioned. The previous held opinion by the Miller (1994) review concerning potential complications from ECT during pregnancy to be minimized by improved technique, are also questioned by our results.

### Check lists

The study by Salzbrenner et al. (2011) provides a 10-point checklist for pregnant women undergoing ECT. Similarly, a 14-item list for general measures and routine anesthetic measures in order to avoid gastric reflux is provided by O’Reardon et al. (2011). The need for close clinical collaboration between gynecology/obstetrics, anesthesiology and psychiatry together with clear responsibility is evident. Textbook checklists for when ECT is an option during pregnancy (Stewart and Erlick Robinson 2001) need updating of potential risks to be considered.

Our results reveal that all potential risk arising from the complexity of ECT intervention, the grand mal seizure, anesthetic and concomitant or previous psychotropic medications, is of great concern and must be taken into account for both mother and fetus/child, and weighed against the clinical benefits, when deciding to administer ECT during pregnancy.

#### Ethical issues

Ethical considerations and possible ethical violations for both mother and the unborn non-consenting child are not discussed. Conflicting opinions can easily arise, such as that described by Polster and Wisner (1999) where the obstetrician advised that ECT be discontinued after premature labor treatment in the obstetrics unit, but ECT was continued by the psychiatric unit. All arguments from this review support the need for holistic clinical decision making and caution when ECT is considered as an option during pregnancy.

#### Strengths and limitations

The strength of this paper is the thorough, systematic review of all published literature without any data limitation. Data extracted from the included studies have strictly been limited to primary case presentations by the authors and not secondary “known to the authors” numbers referred to by the authors in the body text. Likewise all other literature review studies on the subject without any primary case data have also been excluded. The most consistent findings in all included studies was the number of ECTs administered, thereafter the diagnostic indication, pregnancy length, ECT parameters, anesthesia type, condition of both mother and child, the latter was somewhat more dependable in newer date studies. The strength of case study design is the reporting of rare and adverse events, however limitations as for this design must clearly be taken into account.

A limitation is uncertainty in the very oldest published cases, where case presentation is mixed with cases “known to authors” in the manuscript text, to completely document all cases since the introduction of ECT in 1938. The earliest published case reports are also much more likely to be mixed with other treatment forms, such as insulin coma, which is not used and out of date today and these mixed treatment reports therefore not so relevant for today’s practice. No prospective or controlled study design of ECT in pregnancy are found, case studies alone in this field provide the knowledge background. Case studies are susceptible to reporting and publication bias, and only descriptive aggregation of study data is possible, no meta-analyses. As cases of ECT during pregnancy where the treatment went well are most likely not published, the included studies in this review might very well be over represented with adverse event reporting.

#### Clinical implications

ECT during pregnancy should be a last resort treatment. For example in cases of severe depression, catatonia, medication resistant illness, extremely high suicide risk, psychotic agitation, severe physical decline due to malnutrition or dehydration or other life threatening conditions (for example malignant neuroleptic syndrome), where other treatment options are not possible or very inadequate. All potential risks of the ECT treatment, taking into account both mother and fetus, should be weighed against benefits. The ECT should be administered in a hospital emergency setting or delivery room. Information to patients of all possible risks involved should be considered compulsory. ECT during pregnancy should be administered by a highly skilled and competent specialist team consisting of psychiatrist, gynecologist/obstetrician, and anesthesiologist. Monitoring of patient under ECT treatment and also in the recovery room should include midwife and psychiatric nurse. The establishment of a multi-disciplinary specialist team bearing full treatment and follow-up responsibility is fundamental for the safety of the intervention.

## Conclusions

Case reports on ECT administered during pregnancy provide vital knowledge. ECT during pregnancy is advised considered only under very stringent diagnostic and clinical indications, weighing all potential risks against benefits. Updated clinical guidelines are urgently needed in this field.

## Appendix 1

**Table 5:** Search strategy in 2010

	Ovid MEDLINE(R) 1946 to September week 3, 2010	EMBASE 1974 to 2010 week 38	PsycINFO 1806 to September week 4, 2010	Wiley, Cochrane Library, Issue 3 of 4, Jul 2010	Ovid nursing database 1950 to September Week 3 2010	EBSCO; Cinahl, October 2010
1	Electroconvulsive therapy/	Electroconvulsive therapy/	Exp electroconvulsive shock/	MeSH descriptor electroconvulsive therapy explode all trees	Electroconvulsive therapy/	S5 and S10
2	(Electroconvulsive$ or electr$ convulsive$).tw.	(Electroconvulsive$ or electr$ convulsive$).tw.	(Electroconvulsive$ or electr$ convulsive$).tw.	(Electroconvulsive* or electr$ convulsive*):ti,ab	(Electroconvulsive$ or electr$ convulsive$).tw	S6 or S7 or S8 or S9
3	(Electroshock$ or electr$ shock$).tw.	(Electroshock$ or electr$ shock$).tw.	(Electroshock$ or electr$ shock$).tw.	(Electroshock* or electr* shock*):ti,ab	(Electroshock$ or electr$ shock$).tw.	TI (pregnan* or gravid* or gestation*) or AB (pregnan* or gravid* or gestation*)
4	ect.tw.	ect.tw.	ect.tw.	ect:ti,ab	ect.tw.	TI (antenatal* or prenatal* or perinatal*) or AB (antenatal* or prenatal* or perinatal*)
5	or/1–4	or/1–4	or/1–4	(#1 OR #2 OR #3 OR #4)	or/1–4	(MH “expectant mothers”)
6	exp pregnancy/	exp pregnancy/	exp pregnancy/	MeSH descriptor pregnancy explode all trees	exp pregnancy/	(MH “Pregnancy+”)
7	Pregnant women/	exp “parameters concerning the fetus, newborn and pregnancy”/	exp pregnancy outcomes/	MeSH descriptor pregnant women explode all trees	Expectant mothers/	S1 or S2 or S3 or S4
8	(Antenatal$ or prenatal$ or perinatal$).tw.	(Antenatal$ or prenatal$ or perinatal$).tw.	Prenatal exposure/	(Antenatal* or prenatal* or perinatal*):ti,ab	(Antenatal$ or prenatal$ or perinatal$).tw.	AB ect or TI ect
9	(Pregnan$ or gravid$ or gestation$).tw.	(Pregnan$ or gravid$ or gestation$).tw.	(Antenatal$ or prenatal$ or perinatal$).tw.	(Pregnan* or gravid* or gestation*):ti,ab	(Pregnan$ or gravid$ or gestation$).tw.	TI (electroshock* or electr* shock*) or AB (electroshock* or electr* shock*)
10	or/6–9	or/6–9	(Pregnan$ or gravid$ or gestation$).tw.	(#6 OR #7 OR #8 OR #9)	or/6–9	TI (electroconvulsive* or electr* convulsive*) or AB (electroconvulsive* or electr* convulsive*)
11	5 and 10	5 and 10	or/6-10	(#5 and #10)	5 and 10	(MH “electroconvulsive therapy”)
12			5 and 11		From 11 keep 1–11

**Table 6:** Search strategy, update in 2012

	Databases Ovid (federated search): British Nursing Index (1985 – December 2012); Embase (1974 – 2012 December 18); Ovid MEDLINE(R) (1946 – Present); Ovid Nursing Database (1948 – December week 2 2012); PsycINFO (1806 – December week 2 2012)	Wiley, Cochrane Library December 2012	EBSCO; Cinahl, December 2012	SveMed, December 2012	ISI web of Knowledge (SCI-EXPANDED, SSCI, A and HCI.)
1	(Search strategy and search terms the same for all databases as in Table 1)	(Search strategy and search terms the same for all databases as in Table 1)	(Search strategy and search terms the same for all databases as in Table 1)	Electroconvulsive therapy	Topic=(Electroconvulsive Therapy or electroshock* or “electr* shock”*) AND Topic=(pregnan* or gestation* or gravid* or antenatal* or prenatal* or perinatal*)
2					Timespan= 1975–2012

## Appendix 2

**Table 7:** Excluded studies (*N* = 33)

First author (year published)	Comments and reason for exclusion: (1) about ECT, but not in pregnancy, e.g., in postpartum or other conditions; (2) commentary, no primary data, too sparse data, review without primary data, letter to editor; (3) parallel other language publication, not possible to find or full text retrieve; and (4) not relevant topic, about anesthesia types or other topic, e.g., electrical shock injury in pregnancy
Bader et al. (2010)	(2) No study data
Passov (2010)	(2) Conference abstract about 2 cases of ECT in pregnancy, insufficient data
Pinette and Wax (2010)	(2) Letter to editor, without study data
Anderson and Reti (2009)	(2) Literature review, not primary study
Nielsen et al. (2007)	(2) Literature review, not primary study
Richards (2007)	(2) Editorial, not primary study
Maletzky (2004)	(1) About ECT, but not pregnancy
Ginsberg (2007)	(2) Commentary about another article by Pinette et al. (2007)
Howe and Srinivasan (1999)	(1) About Cotard’s Syndrome, ECT given in postpartum after delivery by cesarean section
Berle (1999)	(1) Four cases of severe postpartum depression, ECT given in postpartum
Cutajar et al. (1998)	(1) Case of severe depression in young woman with mild learning disabilities, given ECT in the post-partum period
Ratan and Friedman (1997)	(1) About Capgras syndrome in puerperium, ECT given in postpartum period
Anonymous (1997)	(2) Editorial commentary, no primary author, about electrical shock injury
Johnson (1996)	(1) Case of mania in pregnancy, ECT given in postpartum period
Finnerty et al. (1996)	(1) Case 33 years, pregnant (para 3) with bipolar disorder. ECT was planned given during pregnancy but due to spontaneous rupture of membranes and Caesarian section at 29 gestation weeks (baby reported ok), ECT was administered in postpartum period.
Bernardo et al. (1996)	(1) Imaginary pregnancy, not pregnant
Bruggeman and de Waart (1994)	(2) Letter to editor about another article
Eskes and Nijhuis (1994)	(2) Commentary to case study by Verwiel et al. (1994)
Yoong (1990)	(4) Not about ECT, but electrical shock injury and baby died 24 hours after delivery
Kramer (1990)	(2) Letter to editor about use of ECT in pregnancy
Sneddon and Kerry (1984)	(1) 55 cases of puerperal psychosis treated with ECT in postpartum
Raty-Vohsen (1982)	(4) General treatment of postpartum psychoses
Levine and Frost (1975)	(4) Only about anesthesia type and cardiovascular responses to ECT in 3rd semester pregnancy
Remick and Maurice (1978)	(2) Letter to editor, without study data
Cohn et al. (1977)	(1) About postpartum
Protheroe (1969)	(1) Puerperal psychoses follow-up study and ECT given in postpartum
Anderson (1968)	(2) Dissertation abstract
Marcelino Da Silva and Alexandre (1950)	(3) Not able to retrieve/find
Impastato and Gabriel (1957)	(1) About ECT in postpartum
Forssman (1955)	(4) Not relevant topic, only information on follow-up of 16 children whose mothers were given ECT in pregnancy between years 1947 to 1952
Forssman (1954)	(3) Parallel publication in Swedish to English article of later date by Forssman (1955)
Stone and Walker (1949)	(4) Article not human (rats) study data
Walker (1992)	(3) Same clinical case presented as in article by Livingston et al. (1994)

## Appendix 3

**Table 8:** Summary of findings tables of included case studies *N* = 67 (sorted descending by year)

Primary author and year	Study type: Case(s) Number (*N*) Country	Background Age in years Para pregnancy number (P), Gestation weeks (GW), Diagnoses, rating scales (e.g., Hamilton Depression (HDRS)), Medication, etc.	ECT parameters Number of ECTs, treatment frequency, electrode placement bilateral (BL) or unilateral (UL), Brief pulse or sine wave current, device, etc.	Anesthesia and monitoring Anesthesia, Oxygenation, monitoring of mother (patient) and fetus (fetal heart rate (FHR)), etc.	Mother comments and adverse events Vaginal bleeding, Uterine contractions, Abdominal pain, Premature labor, Miscarriage, Meconium-stained amniotic fluid, etc.	Fetus, baby/child comments and adverse events FHR in beats per minute (bpm), fetal cardiac arrhythmias, and fetal malformations Stillbirth, neonatal death, neonatal respiratory distress, etc.	General comments and treatment efficacy Postpartum treatment, symptom remission or relapse, other information, etc.
De Asis et al. (2013)	Case USA	20 years, P2, GW 23 Bipolar disorder (6 year history) Patient requested ECT due to previous termination of pregnancy and fear of teratogenic effects of medication	14 ECTs (given from 23 to 39 GW) Right UL Device: Mectra Spectrum 5000Q	Anesthesia: methohexital and muscle relaxant succinylcholine for first 2 ECTs and then changed to propofol for all next ECTs	On 2nd ECT at 24 GW, prolonged seizure duration 201 s and fetal heart deceleration (profound bradycardia) after 120 s. Medazolam given to stop seizure. Emergency cesarean delivery prepared, but not undertaken when FHR normalized	Baby delivered at full term Apgar 10	Anesthetic agent changed from methohexital to propofol due to serious FHR deceleration
Gahr et al. (2012)	Case Germany	35 years, P1, GW 4 (at admission) Recurrent depressive disorder (6 year history) Treated with Fluoxetine (20 mg/day) last 2 years. rTMS addon therapy to fluoxetine for 5 weeks during pregnancy did not respond to 24 sessions of rTMS [5 rTMS sessions/week, frequency = 15 Hz; intensity = 110 % of resting motor threshold (40 % of max. stimulator output)	15 ECTs (started at 14 GW) Right UL, 3 times weekly Device: Thymatron DG ECT unit, Somatics, LLC. Stimulus intensity between 30 and 65 % of max. stimulator output. Seizure duration 21–32 s	Anesthesia: Alfentanil augmented with propofol without the use of volatile anesthetics. Muscle relaxant succinylcholine. 100 % oxygenation Monitoring: sonographic fetal control Mother: Magnetic resonant imaging (MRI) scan of the brain normal (before ECT)	After 24 GW no more information about mother	No report of fetal trauma up to 24 GW After 24 GW no information about fetus/baby	Remission of symptoms by Beck Depression Inventory scores from 56 (before ECT) to 4 (1 week after last ECT)
Yang et al. (2011)	Case South Korea	33 years, P1, GW 28 Schizophrenia History of 15 years schizophrenia, hospitalized 5 times due to psychotic symptoms. Medicated with risperidone, benzotropine, zolpidem, trazodone, quetiapine before admission. Olanzapine also taken	7 ECTs during 2 weeks 168mC seizure 75 s Patient in tilt position with pad under right side hip	Anesthesia: Thiopental 4 mg/kg and muscle relaxant succinylchlorine 1 mg/kg, 100 % oxygenation Monitored with electrocardiography, pulse oxymetry, blood pressure. FHR and uterine contractility by ultrasound under and after ECT	1 h after 1st ECT session uterine contractions, regarded as pre-term labor. Tocolytic treatment with 50 mg ritodrine and 500 ml intravenous dextrose. Emergency caesarian section at 35 GW, 3 weeks after last ECT	FHR variability 140–160 bpm under ECT. Baby premature, 1,940 g Hyaline membrane congenital disease and hypertrophic pyloric stenosis	Baby at 2 months operated with pyloromyotomy procedure
O’Reardon et al. (2011)	Case USA	39 years, P3 (previous twins), 20 GW Severe depression, psychomotor agitation, dysphoric. HAM-D24, BDI 48, BAI 50, non-responsive to antidepressant medication (sertraline, paroxetine plus quetiapine augmentation). Graves disease, treated with propylthiouracil. Previous major depressive episodes 6 and 4 years before current. 1st episode postpartum onset, 2nd during twin pregnancy resulting in elective caesarian delivery	18 ECTs, started in 21 GW on a outpatient basis Last prenatal ECT (number 18) at 35 GW BL bifrontal Device: MECTA Spectrum 5000Q	Anesthesia: methohexital and succinylcholine. Cricoid pressure applied to reduce risk of aspiration. From 15th ECT and onwards, in the 3rd trimester, aspiration risk reduced by oral sodium citrate and intravenous ondansetron and metoclopramide. FHR monitoring before and after ECT with Doppler monitor until GW 30. Patient monitoring with tocometry for uterine activity	Caesarian section (due to 2 previous caesarian deliveries) at 37 GW (2 GW after last ECT) Patient developed small left sided pneumothorax during delivery	Baby girl, 6 lb 7 oz. Apgar scores normal. Child followed up for 18 months, normal development – language, fine motor and social developments within normal limits – no developmental delays	Improvement after 3 ECT sessions, HAM-D24 score reduced from 40 to 20 with similar changes in other scores. 13 continuation ECTs administered in postpartum period over 6 months, thereafter pharmacotherapy for depression and anxiety ECT commented as safe. Provides a list of recommendations for ECT during pregnancy
Salzbrenner et al. (2011)	Case USA	48 years, P1, GW 32 Severe bipolar depression, suicidal. History of hypothyroidism, obesity, hypertension, diabetes mellitus. In vitro fertilization (IVF)	9 ECTs BL ECT given 3 times weekly Brief pulse wave Device: MECTA spectrum 5000Q ECT stopped after 9th session due to cognitive decline	Anesthesia: Methohexital and succinylcholine Also hypertensive medication with labetalol until 6th ECT, thereafter replaced with remifentanil due to increased blood pressure after ECT	FHR monitored. Caesarian section at 38 GW and 6 days, due to preeclampsia and breech presentation	No birth/Apgar data. Child examined at 4 and 9 months, and development reported as normal	Conceived via IVF with donor egg. Postpartum prophylactic oral medication (lithobid) to avoid mania symptoms. Provides a 10 point checklist for pregnant women undergoing ECT
Lovas et al. (2011)	Case Hungary	31 years, P1, GW 7–22 Bipolar disorder History of severe mania Medicated with quetiapine 750 mg/d, diazepam 10 mg/day at GW 6, haloperidol given for 5 days. ECT given due to persistent severe manic and psychotic symptoms	21 ECTs 2 series, 7 given 2 times weekly and 14 given 1 time weekly BL Device: Siemens Konvulsator 2077s. Intermittent current. Not intubated for the first 15 ECTs. Last 6 ECTs ranitidine 20 mg, metoclopramide 20 mg	Anesthesia: Propofol and suxamethonium. Pre- oxygenization. In last 6 ECTs rapid sequence induction anesthesia technique used. Monitoring: Electrocardiography, blood pressure and arterial oxygen saturation. Regular ultrasound examination of fetus	Abdominal pain in 4th ECT session. Caesarian section at 39 GW due to development of preeclampsia symptoms	Baby boy, Apgar 9.	Medication: Quiatipine and lamotrigine medication in 3rd trimester. Cardiotocography not used, since authors claim information from this to be limited before 24 GW
Pesiridou et al. (2010)	Case USA	33 years, P3, GW 30–32 Bipolar II, alcohol and cocaine abuse, borderline personality disorder	6 UL Brief pulse ECT Maternal position: left hip lateral tilt Device: Mecta spectrum 5000Q 60-Hz 15 s seizures first then etomidate substitution increased to 38–45 s	Anesthesia: Methohexital 170 mg and muscle relaxant succinylchlorine 100 mg	10 h after ECT session 6 painful contractions, further intermittent contractions until spontaneous birth at 37 GW	Baby ok Apgar 9	
Serim et al. (2010)	Case Turkey	16.5 years, P1, GW 29 (at admission), GW 31 (at ECT start) Major depression with psychotic features (HDRS score 32)	10 ECTs (lasting 30 s or more) BL (bitemporal) Brief pulse wave Device: Thyamtron System IV	Anesthesia. Propofol 1 mg/kg and muscle relaxant rocuronium. Mask oxygenation. Fetal monitoring: Ultrasonography Examination weekly during pregnancy by obstetrician	After 5th ECT patient improved (HDRS 8). Two weeks after 10th ECT psychotic and depressive symptom relapse. Uterine contractions after one ECT session for 2–3 min in need of tocolytic treatment by obstetrician. FHR decreased to below 120 bpm in 2–3 s during one ECT session. Caesarian section chosen for safe delivery due to mental condition of patient in GW 39	Baby, 1 and 5 min Apgar 10. No abnormality in neonatal examination	Mother treated with antipsychotics and antidepressant (risperidone and paroxetine) during pregnancy and after delivery. Post partum symptom improvement (HDRS 11)
Molina et al. (2010)	Cases *N* = 2 Spain	Case 1: GW 26 Case 2: GW 38 Manic depressive psychosis refractory to medication treatment	13 ECTs altogether for both 2 cases. Frequency, 2 ECTs per week. ECT device not specified	Anesthesia not specified. Cardiotocogram monitoring. Uterine contractions reported after 5 ECTs, disappearing after 58 min (not specified to which case)	FHR decline under 6 ECTs (not specified to which case). Spontaneous delivery at 39 GW (Case 1) and 40 GW (Case 2)	Babies ok, adequate weight. Apgar 9/10 for both	Congress abstract with limited information
Kucukgoncu et al. (2009)	Case Turkey	No age, P or GW data. Schizophrenia Also treated with Clozapine during pregnancy	No data	No data	No adverse effects for the patient	No adverse effects for the baby	Conference paper with sparse data
Ghanizadeh et al. (2009)	Case Iran	30 years, P1, GW 8 Bipolar mood disorder. History of mental illness 12 years. Carbamazepine 200 mg/day taken 5 months prior to pregnancy	9 ECTs total (given between 8 to 12 GW)	Anesthesia: Thiopental 4 mg/kg and muscle relaxant succinylcholine 1 mg/kg Ultrasonography examination - no pathological findings and gestational age 12 weeks and 2 days	Moderate vaginal bleeding after 3rd ECT, lasting 12 h. Given 6 more ECTs, improved and discharged. No uterine contractions or pain. Relapse 20 days later, readmitted manic and given 3 ECTs given in 1 week	No data about fetus, delivery or baby Pregnancy followed only to 12 GW+ 2 days	ECT administered in early pregnancy. Vaginal bleeding after each ECT session and ECT stopped
Malhotra et al. (2008)	Cases *N* = 2 India	Case 1: 24 years, GW 24 Severe depression, suicidal. Case 2: 22 years, GW 26 Catatonia	Case 1: 3 ECTs Case 2: 3 ECTs	Premedication 2 h prior to ECT with ranitidine, metoclopramide and isoxsuprine. Preoxygenated for 3 min with 100 % oxygen. Anesthesia: Thiopentone and muscle relaxant succinylcholine, tracheal intubation. Monitoring fetus: fetal cardiometry. Monitoring patient: heart rate, blood pressure, pulse oximetry, electrocardiogram end-tidal CO_2_. Nursed in left lateral position in recovery room after ECT	and given profylatctic tocolytic treatment with isoxsuprine 10 mg 8 hourly for 48 h	No data	Beyond 1st trimester tracheal intubation preferred to avoid pulmonary aspiration. Mainly about anesthesia, other data very sparse and lacking
Ceccaldi et al. (2008)	Case France	28 years, P1, GW 26–30 (2nd trimester) Bipolar disorder with severe depressive episode. History of bipolar disorder since 16 years old. Venlafaxine and paroxetine medication stopped due to pregnancy	10 ECTs (in 26–30 GW)	Anesthesia: etomidate, propofol and muscle relaxant suxamethonium. Monitoring of FHR	ECT discontinued after 10th ECT due to premature delivery threat. Treated withfluoxetine in month prior to vaginal delivery under epidural analgesia	Delivery at 36 GW. Baby girl healthy, 3,120 g. Neurological examination of child revealed no abnormality	Clinical improvement from ECT reported
Bozkurt et al. (2007)	Case Turkey	34 years, P2, GW 13 Psychotic depression. History of 3 years prior psychotic depression, treated with antidepressant and antipsychotic medication	13 ECTs (3 times weekly) given in one month and 3 ECTs monthly for maintenance until 32 GW before birth. Bifrontal ECT Device: Mecta Spectrum 5000Q	Anesthesia: Thiopental 250 mg, 100 % oxygenation. Airway and cricoid pressure used (not intubated). No lateral tilt used. Patient monitored with blood pressure, electrocardiography	Mother pelvis pain after 8th and 9th ECT. Vaginal delivery at 38 GW	FHR reduced to 90 bpm after 13th and 16th ECT, rose to baseline after 2–3 s. Healthy baby boy at 38 weeks	HDRS score reduced from 33 to 7 (at 10th ECT) and to 3 at release from hospital. Photo of baby boy in article
Kasar et al. (2007)	Case Turkey	32 years, P2, GW 32 Major depressive disorder with psychotic features and suicidal ideation (HDRS 47, IQ 71). Venlafaxin and quetiapine medicated Similar complaints in 1st pregnancy, but not treated then	4 ECTs (frequency 3 ECTs per week) Bifrontal placement Device: Thymatron system IV (Somatics, Lake Bluff, IL)	In 4th ECT anesthesia: Propofol 1 mg/kg and muscle relaxant succinylcholine. Fetal monitoring by obstetric consultations and ultrasonography	1 day after 4th ECT uterine contractions/birth pains – premature labor and caesarian section performed at 34 GW	Baby premature healthy, 2,600 g. Baby: ‘normal’ development for 6 months	After 3rd ECT, improvement in depression, HDRS 15
Pinette et al. (2007)	Case USA	22 years, P1, GW 20–34 Bipolar depression (long history). Prior to pregnancy maintenance ECT treatment	7 ECTs in 20–34 GW Bifrontal ECT every 2nd week in entire pregnancy	No data	Preeclampsia development: elevated blood pressure and urine protein level. Induced labor, vaginal delivery at 36 GW	FHR recorded after each ECT with no abnormalities. Baby boy, 2,550 g 1 and 5 min Apgar scores, 4 and 7. Baby: small left cerebellum and bi-hemispheric deep white matter cortical infarct	Sparse ECT data. Long term motor control issues assumed for baby
Espínola-Nadurille et al. (2007)	Case Mexico	22 years, GW 21 Schizophreniform catatonic features. Haloperidol 5 mg intramuscular injection given in emergency room resulting in malignant catatonic syndrome and acute renal failure	10 ECTs given 3 times weekly with 20 % stimulus BL Device: Thymatron DGx, Also treated with Lorazepam after ECT	Obstetric ultrasonography monitoring of fetus during pregnancy	No data	No adverse effects on fetus observed	Partial remission of symptoms after ECT and further treated with clozapine
Prieto Martin et al. (2006)	Case Spain	35 years, GW 30 Severe depression ECT indication: clinical condition worsened after initiation antipsychotic and antidepressant medication (mirtazapine, fluvoxamine, alprazolam, quetiapine)	9 ECTs (3 times weekly) begun at 32 GW Brief pulse wave Device: Thymatrone TM Somatics Inc	Anesthesia: propofol and succinylcholine with endotracheal intubation Patient and fetus were monitored. No significant variations in maternal blood pressure or heart rate, nor FHR	Tocolytic treatment given when uterine contractions detected after ECT. 2 days after last ECT in 35–36 GW the patient went into premature labor. Vaginal delivery	After 6th ECT FHR deceleration observed. Baby boy, 2,320 g, Apgar 9 after 1 min, Apgar 10 after 5 min	Patient improved from ECT and discharged with only lorazepam medication
Balki et al. (2006)	Case Canada	31 years, P1, GW 22 Bipolar disorder, suicidal Medication: lithium, paroxitene, lorazepam. Lithium discontinued and other medication continued during pregnancy	1 ECT (with 3 successive electrical current stimulations given). Right UL	Anesthesia: Thiopental 250 mg and muscle relaxant succinylcholine 100 mg. Endotracheal intubation. 40 % oxygenation. Patient monitored with electroencephalogram (EEG). MRI scan of brain taken showing increased signal over parietal area consistent with seizure activity. FHR monitored intermittently by obstetrician	After last 3rd ECT stimulus continuous grand mal seizures occurred. In attempt to stop seizure given large doses thiopental, diazepam and propofol over 2½ h. Followed by thiopental and propofol infusion. EEG demonstrated seizure activity for 5 h. Patient transferred to intensive care unit. Due to hypotension treated with phenylephrine and dopamine infusion. On 7th day patient regained consciousness and extubated. EEG mild encephalography	On 2nd day fetus died, labor ensued and spontaneous vaginal delivery on 3rd day	Patients ICU complicated with diabetes insipidus, renal and left ventricular dysfunction
Maletzky (2004)	Cases *N* = 4 USA	Case 1: 27 years, GW unknown, MDD 2 months after pregnant 2 Cases Major depressive disorder (MDD) 2 Cases MDD with psychotic features	Case 1: 6 ECTs, BL, over 2 weeks Case 2: 8 ECTs Case 3: 5 ECTs Case 4: 8 ECTs Device: Mecta Spectrum	No data	No data	Case 1: healthy boy baby Cases 2–4: no data	Case 1: Post partum ECT due to relapse of symptoms 4 weeks after delivery, response to ECT good at both time points Only one out of 4 pregnancy cases reported with more detail
Brown et al. (2003)	Case USA	37 years, P1, GW 20 Psychotic depression	8 ECTs during 3 weeks Position, left uterine displacement	Preoxygenation Anesthesia: Thiopental 3 mg and succinylcholine 1.6 mg/kg. Intubation difficulties in 1st ECT due to mandibular, teeth and palate anatomical condition. ProSeal^TM^ LMA chosen for airway management during all further ECTs	No adverse events reported	No data	A case report concerned more with the airway management and prevention of aspiration
DeBattista et al. (2003)	Case USA	41 years, P1, 17 GW Major depression, withdrawn from daily nefazodone medication at approx. 4 weeks gestation	5 ECTs BL Brief pulse wave Device: Thymatron. Device set at 45 % maximum for all ECTs	Anesthesia: Thiopental (in first 2 ECTs), etomidate (in last 3 ECTs) with muscle relaxant succinylcholine, 100 % oxygenation. Premedication with bicitra per os and intravenous metoclopramide to avoid gastric reflux. Maternal electrocardiogram, blood pressure monitoring and EEG during ECT. FHR monitored with Doppler before and after ECT. Lateral tilt not used	Maternal heart rate and blood pressure increase 20 % Vaginal delivery at 38 GW	In 4th ECT FHR deceleration down to 100 bpm In 5th ECT FHR deceleration down to 60 bpm, lasting 3–5 s. Baby boy, 38 weeks, ok	HAM-D score reduced from 31 pre ECT to 7 post ECT and patient discharged
Fukuchi et al. (2003)	Case Japan	36 years Obsessive compulsive disorder (OCD) Pharmacotherapy ineffective	2 ECTs	Anesthesia given but type unknown. Monitoring: cardiotocography throughout the procedure FHR decline during 2nd ECT	Uterine contractions after 2nd ECT, tocolytic treatment with ritodrine. No delivery data	No baby data	Only abstract data, due to Japanese language
Iwasaki et al. (2002)	Case Japan	24 years (GW> 26, in 3rd semester) Schizophrenia (10 year history) treated with oral antipsychotics	6 ECTs BL, alternative current (sine wave)	Anesthesia: thiamylal and suxamethonium 100 % oxygenation At 6th ECT general anesthesia maintained by sevoflurane in oxygen, followed by suxamethonium	Monitoring: Maternal hemodynamic variables, arterial oxygen saturation (Spo2), uterine contractions by cardiotocogram At 3rd ECT continuous uterine contraction refractory to tocolysis for 6 min resulting in fetal bradycardia AT 6th ECT uterine contraction diminished Monitoring of FHR	3rd ECT fetal bradycardia 6th ECT FHR unchanged	Only abstract data, due to Japanese language
Iwasaki et al. (2002)	Case	31 years, GW 21 (P unknown) Depression	14 ECTs over 65 days	Anesthesia: thiamylal or propofol. Propofol chosen when severe nausea after thiamylal. Patient laid in a supine position during ECT	FHR monitoring: significant decrease in FHR with propofol, none with thiamylal	Delivered healthy baby, 3 years old and well	Patient gradually improved after ECT. Very brief report with sparse data
Polster and Wisner (1999)	Case USA	29 years, P1, GW 26 Paranoid schizophrenia with depressive symptoms History of 2 years treatment with risperidone and paroxetine. Patient self discontinued medication before pregnancy. Became increasingly psychotic, treated with risperidone in 23 GW for 19 days. Increasingly depressed, suicidal, catatonic and little effect from loxapine, lorazepam and nortriptyline. ECT indication “medication resistant”	12 ECTs, 3 times weekly (total course lasting 3 ½ weeks) 8 right sided UL and 4 BL, BL after 8th ECT Prophylactic preterm labor treatment with terbutaline and indomethacin in 2nd to 12th ECT	Anesthesia: 240 mg thiopental and muscle relaxant 80 mg succinylcholine. Additional 80 mg thiopental given in order to discontinue seizure. Obstetric nurse monitored FHR before, during and after ECT	After 1st ECT uterine contractions every 2–3 min. Premature labor, tocolytic treatment with indomethacin and ritodrine. Trichomoniasis infection of urinary tract treated with metronidazole and nitrofurantoin. During 12th ECT transient, patient had significant bradycardia and hypoxemia. ECT stopped	No data	Obstetrician advised ECT discontinued after premature labor treatment in obstetric unit, but ECT was decided continued by psychiatric unit. ECT discontinued due to minimal improvement
Gilot et al. (1999)	Case France	28 years, GW 20 (at admission), GW 28 at ECT start Severe depressive disorder, with agitation and psychosis History of 8 years recurrent mood disorder. Treated with clomipramine and phenothiazine. Also amitriptyline, haloperidol, oxazepam and nitrazepam. ECT decided after 7 weeks due to lack of medication response	9 ECTs in 5 weeks BL Sinus wave Left lateral tilt Improvement observed after 9 ECTs	Anesthesia: Propofol, 100 % oxygenation and oral-tracheal intubation Monitoring: Ultrasonography, recording of uterine contractions and FHR	FHR change observed during anesthesia. Fetus examination at 32 GW as normal. At 34 GW, signs of fetal ascitis on routine ultrasonography. Emergency caesarian section	Baby boy, Apgar score 8 and 9. Immediate surgical treatment for vascular meconium peritonitis. Ascitic fluid sterile, no bacteria or virus found. Baby died 9 days later, due to metabolic post-surgical complications. Examination of baby revealed perforation of the sigmoid colon, and a left temporal sub-dural hematoma. Probable cause of death anoxic-ischemic in nature	ECT administered in a surgical-obstetric environment. Multidisciplinary discussion between Psychiatrists, anesthetists and obstetricians for ECT indication
Bhatia et al. (1999)	Cases *N* = 2 USA	Case 1: 26 years, P1, GW 35 (at admission) GW 37 (at ECT start) Recurrent major depression (last episode started at 15 GW). Also dysmorphophobia and OCD thinking patterns. Treated with desipramine, lorazepam and loapine succinate at GW 35 for 2 weeks before ECT. History of 5 years, multiple admissions and imipramine medication without sufficient effect. Case 2: 23 years, P4, GW 27 (at admission) GW 28.7 (at ECT start) Generalized anxiety with panic attacks. Treated with desipramine, oxazepam and tryptophan without sufficient response. History of 8 years generalized anxiety with panic attacks	Case 1: 6 ECTs (from GW 37 to 39) 3 times weekly BL Case 2: 6 ECTs BL	Case 1: Anesthesia: Thiamylal, succinylchlorine and curare. 100 % oxygenation and intubation. Monitoring: pelvic examination, tocodynamometry and FHR. Case 2: Anesthesia: Methohexital and succinylchlorine. 100 % oxygenation and intubation. 67 s seizure after 1st ECT. Monitoring: After 6th ECT (GW 31) preterm labor contractions	Case 1: uterine contractions after 2nd ECT. After 3rd ECT tocolytic treatment. After 6th ECT uterine contractions lasting 12 h post ECT and transferred to maternity ward. FHR variability during uterine contractions and decreased in 3rd ECT. Case 2: No FHR variability or uterine contractions until after 6th ECT. Post ECT preterm labor (at 31 GW) subsided with tocolytic treatment	Case 1: after 6th ECT absence of fetal movement for 25 min. Healthy girl baby 6 lb 4 oz (2,835 g), born at 39 GW (2 days after last ECT and after being discharged home) Case 2: healthy baby boy, 7 lb (3,175 g) born at 35 GW	ECT administered in delivery room. Both patients mental status reported improved after ECT series. At follow-up 6 months after ECT both patients symptom free.
Echevarria et al. (1998)	Case Spain	25 years, GW 8 Reactive depression and delusional disorder	3 ECTs (ECT given every 2nd day) BL Sine-wave current Device: Siemens Konvulsator 2077-S 1st ECT seizure duration 17 s, 2nd 24 s, 3rd 22 s	Anesthesia: Premedication 0.01 mg/kg Atropine. Pre-oxygenated 100 % oxygen for 2 min. Thiopental 4 mg/kg and muscle relaxant succinylcholine 1 mg/kg. Monitoring: electrocardiogram, blood pressure and pulse oximetry. Ultrasonograms before and after ECT	After 2nd ECT vaginal bleeding. After 3rd session profuse vaginal bleeding. Miscarriage 4 h later	After 3rd ECT miscarriage	After miscarriage Patient received 6 more ECTs discharged in complete clinical remission
Livingston et al. (1994)	Case twins *N* = 2 USA	28 years, P1, GW 26–34 Severe depression. At admission confused, suicidal, violent, not eating and delusional. Medication prior to ECT: nortriptyline, perphenazine, fluoxetine, thiothixene, benzotropine mesylate. History of 3 years depression, treated with lithium, thiothixene, benztropine mesylate, fluoxetine, nortriptyline – having received some of these drugs in early pregnancy	8 ECT sessions Minimal bipolar setting used for generating 60–90 s seizures	Anesthesia: endotracheal intubation Left lateral tilt position. Monitoring: electrocardiography, EEG, pulse oximetry. Uterine activity and FHR also	Spontaneous preterm labor at 35 GW	FHR deceleration for 2.5 min after 3rd ECT Twin A, 2,549 g Apgar 6 and 7 Transposition of great vessels. DIED of post operative complications Twin B, 2,894 g Apgar 6 and 8 Anal atresia, small sacral defect, coarctation of aorta	Fetal outcome (death) for one twin infant. Both infants normal 46XX karyotypes. Symptom relapse post partum, treated with ECT and diverse medication
Verwiel et al. (1994)	Case Netherlands	27 years, 18 GW Treated with clorazepate and oxazepam in pregnancy. ECT indication: Malignant neuroleptic syndrome (MNS) after Haloperidol treatment, unresponsive to dantrolene	2 ECTs, given at 29 GW and 3 days, prior to 9 weeks of MNS	Anesthesia: thiopental 125 mg and succinylcholine 35 mg. Monitoring: cardiotocography during ECT and ultrasound fetus every 7 days	On day 88 vaginally delivery without complications after a fever peak of 39 °C with leukocyte count of 23 × 10 g/l and 5 bars in the image differentiation	Baby girl healthy, 1,790 g Apgar score 8 and 9 after 1 and 5 min. Ventilation not needed and no sepsis. Prophylactic antibiotics given, from 2nd day phototherapy (high bilirubin and normal liver function values)	Transferred to another psychiatric ward and discharged after a few weeks in reasonable condition together with healthy daughter
Vanelle et al. (1991)	Cases *N* = 5 France	Case 1: 30 years, P3, GW 20 (4½ months) Bipolar II disorder History of previous depressive episodes and hypomania. Treated with Quinuprine (tricyclic antidepressant) and clomipramine in 1st trimester without effect. Case 2: 32 years, P3, GW 20 (4½ months) Unipolar depression (melancholic) Case 3: 27 years, P2, GW 27 (7 months) Schizoaffective disorder ECT due to melancholic and delusional state. History of postpartum psychoses Case 4: 27 years, P 1, GW 14 (4 months) Schizoaffective disorder ECT due to psychotic anxiety state. Case 5: 28 years, P1, GW 7 (1½ months) Psychotic depression History of melancholy, hypomania previous abortion. ECT given to avoid antipsychotic drugs in early pregnancy	Case 1: 10 ECTs Case 2: 10 ECTs Case 3: 6 ECTs Case 4: 9 ECTs Case 5: 20 ECTs	Anesthesia: Propanidid (Epontol) and muscle relaxant (at low dose to avoid uterine contractions) and oxygenation. No fetal monitoring		Case 1: Full term baby ok Case 2: Full term baby ok Case 3: Full term baby ok Case 4: Full term baby ok Case 5: Fetus death at 11 GW	Case 4: Developed postpartum mania antipsychotic (pipothiazine) medication and mood stabilizer (carbamezapine) Case 5: used lithium and amitryptyline in early pregnancy
Sherer et al. (1991)	Case USA	35 years, P2, GW 30 Psychotic depression	7 ECTs BL temporal lobe ECT frequency, 1 time weekly Device: Thymatron Somatics Inc, Lake Bluff Ill. 30 % stimulus setting (pulsed bidirectional square- wave) fixed pulse 1 s and frequency 70 Hz, 50 s seizures	Anesthesia: Thiopental sodium 125 mg and succinylcholine 50 mg. 100 % oxygen Mother and fetus monitored. At 32 GW Doppler velocimetric monitoring before, during and after ECT	Bleeding and uterine contractions after each ECT Transient hypertension after ECT. At 31 weeks tocolytic treatment with terbutaline. At 34 weeks observation in delivery suite needed due to bleeding. Spontaneous labor 37 GW and caesarian section performed	FHR reduction after 1st ECT Baby boy, 2,704 g Apgar 3 and 9	Large retro-placental clot confirming abruption placentae diagnoses
Yellowlees and Page (1990)	Case Australia	22 years, (P unknown) GW 29 (at admission) GW 32 (at ECT start) Diagnoses somewhat unclear - catatonic features and psychotic depression Antipsychotic medication with Haloperidol 10 mg daily prior to ECT and stopped at 32 GW. Also given a course of amytriptyline	9 ECTs over 3 weeks UL (ECT type noted as low voltage and no other data) ECT administered in surgical recovery room with obstetrician present	Anesthesia: general anesthesia with endotracheal intubation 100 % oxygen Fetal monitoring by cardiotocograph and ultrasound. Maternal oxygenation by oximetry. Maternal oxygenation between 99-100 % saturation	FHR normal	Baby girl born at 37 GW, 3,050 g Apgar 8 and 9. Child examined at 3 months follow-up: “no developmental abnormalities”	Post partum diagnosis: Schizoaffective psychosis, IQ 63 At 3 months follow-up “well” and taking fluphenazine decanoate (25 mg every 3 weeks) and amitryptyline (100 mg at night)
LaGrone (1990)	Case USA	23 years, GW 22–23 Acute mania (agitated, psychotic) and sickle cell anemia. History of cholecystectomy at 19 years. Previous psychiatric admission and antipsychotic medication (thioridazine)	7 ECTs BL Device: Thymatron, Lake Buff, Illinois (Brief-pulse current) 1st seizure induced with 50 % energy, duration prolonged 260 s and aborted with intravenous diazepam. Remaining ECTs at 30 % energy and durations 62–126 s	Anesthesia: Glycopyrrolate, methohexital and succinylcholine with 100 % oxygenation. Intubated each time. External monitoring av fetus	17 days after last ECT relapse of symptoms, readmission and medicated with haloperidol. Premature labor at 34 GW. Delivery by Caesarian section due to genital herpes infection	Baby boy 1,445 g required intubation Apgar 4 and 6 Infant growth retardation	Postpartum symptom relapse, treated with 6 ECTs and haloperidol, then maintained on litium and fluphenazine
Griffiths et al. (1989)	Case USA	30 years, P2, GW 22 (at admission) GW 23 (at ECT start). East Indian woman. Major affective disorder (major depression psychotic type) History of hypothyroidism treated with levothyroxine	11 ECTs total: 6 ECTs in 23–26 GWs and 5 ECTs in 28–31 GW 3 times a weeks Bifrontemporal ECT shock 1.00-1.25 s and current 60Hz with 1.6-msec pulse width. Seizure duration 30–50 s observed in one extremity by arterial tourniquet method	Anesthesia: Pre- medication with glycopyrrolate. Thiamylal sodium and muscle relaxant succinylcholine. Monitoring: Maternal blood oxygen saturation, blood pressure, electrocardiogram and uterine activity. FHR monitoring	Normal parameters for maternal and fetal monitoring. Spontaneous delivery at 40 GW	Baby boy 2,900 g Apgar 9 and 9 at 1 and 5 min	Discharged with thioridazine medication at 31 GW
Mynors-Wallis (1989)	Case UK	28 years, GW 28 Ghanian woman Depression	ECT course (number of ECTs not stated)	No data	No data	No fetus /child data	Letter to editor. Sparse data. Response to ECT reported as good
Varan et al. (1985)	Case Canada	33 years, P1, GW 18–20 Paranoid schizophrenia Long-standing history of psychiatric illness. Chlorpromazine medication in early pregnancy and before entering hospital. Chlorpromazine medication during pregnancy until discharge	12 ECTs total over 24 days. BL first 3 days, then right UL, 3 times weekly. Device: MECTA with minimum effective settings	Anesthesia: Methohexital (Brietal), muscle relaxant succinylcholine and 100 % oxygenation	Monitoring: EEG, electrocardiogram (EKG) and of mother. FHR by Doppler. Transient FHR bradycardia noted in tonic phase of treatment. At 38 GW mild pre eclampsia toxaemia diagnosed. Labour induced at term, normal vaginal delivery. Slight amnesia, minimal anterograde memory impairment, slowing of motor speed- normal after 3 weeks	Baby boy 4,270 g. Apger 9/9. No fetal abnormalities at birth and 8 days follow-up	Discharged 8 days after birth. Psychiatrically post partum stable
Dorn (1985)	Case USA	27 years, GW 8 Bipolar affective disorder Psychotic depression at admission. History of psychiatric hospitalizations since age 20 years. Mild cerebral palsy diagnoses. Bilateral hearing loss since age 5. Small atelectasis of right lower lung lobe but no active pulmonary disease. Haloperidol, benztropine, doxepin medication in early pregnancy – discontinued when discovered pregnant	9 ECTs BL Device: Medcraft B-24 Alternating Current 170 V for 1 s (sine wave type)	Anesthesia: Glycopyrrolate premedication. Methohexital sodium 80 mg and muscle relaxant succinylcholine 80 mg. Ventilation by oxygen mask (no endotracheal intubation). Monitoring: Maternal blood gases before and after ECT. FHR by either Doppler or ultrasonography. Electroenchephalogram (EEG) taken after 5th , 7th and 9th ECT	Maternal blood pressure and pulse increased slightly immediately after ECT but no maternal or fetal heart arrhythmias. FHR 140 bpm after 4th ECT No birth data	No data	Symptoms improved after 6th ECT. After 9th ECT mildly hypomanic. Discharged with outpatient planned maintenance ECT. Obstetrician and anesthesiologist present alongside psychiatric staff during ECT. ECT during pregnancy regarded as safe
Wise et al. (1984)	Case USA	24 years, P2, GW 28 Psychotic depression Antipsychotic medication taken 8 months before pregnancy Nortriptyline medication during pregnancy	12 ECTs UL (non-dominant hemisphere) No ECT type data except “low voltage”. ECT administered in labor and delivery suite. Obstetrician present	General anesthesia and endotracheal intubation. Monitoring: Cuff technique and EEG recordings. Uterine muscle tone by tocodynamometer. FHR by Doppler	Post ECT patient had brief episode of supraventricular tachycardia. No uterine contractions noted after ECT. No abnormal FHR. Oxytocin induced vaginal labor at 37 GW due to sustained hypertension	Baby 7 lb, 6 oz Apgar 8 and 9, at 1 and 3 min	Remission of depressive symptoms after 8 ECTs but then relapse requiring 4 additional ECTs
Repke and Berger (1984)	Case USA	33 years, P2, GW 19.5 (at admission) Severe depression, suicidal. History of 4 years, treated with imiprimine and desimipramin. Medication discontinued when discovered pregnant but started again due to severe condition, given desimipramin up to 200 mg per os twice daily for 30 days with minimal improvement, then ECT	2-5 ECT courses (no other ECT type data)	Anesthesia: Atropine premedication. Methohexital sodium, pancuronium bromide, and succinylcholine chloride. Marked drop in blood pressure after first ECT	FHR transient elevation	Baby 3,024 g Apgar 8–9, normal delivery Baby transient hyperbilirubinemia Baby born 3 months after discharge 3 Neurological examination of baby at 1 month, reported within normal limits	52 days hospital stay
Loke and Salleh (1983)	Cases *N* = 3 Malaysia	Case 1: 21 years, P1, 26+ GW at admission Case 2: 25 years, P2, 26+ GW at admission Case 3: 22 years, P1, 26+ GW at admission Diagnoses: All schizophrenia, DSM-III Medication: Case 1: oral Chlorpromazine 200 mg and Haloperidol 6 mg Case 2: oral Chlorpromazine 50 mg and Haloperidol 4.5 mg Case 3: oral Chlorpromazine 100 mg and 100 mg intramuscular injection when needed	Case 1: 5 ECTs Case 2: 6 ECTs Case 3: 6 ECTs	No data	Case 1: Spontaneous vaginal delivery after ECT Case 2: Breech presentation, delivered at term Case 3: No data about delivery	Case 1: Baby 3.2 kg Apgar 9–10 Case 2: Baby 3.3 kg, Apgar 6–10 No fetal abnormality reported in 2 of cases No data about case 3 baby	Case 2: Postpartum relapse and given 8 ECTs Case 3: 11 years psychiatric history of chronic schizophrenia
Impastato et al. (1964)	Case USA	No age, 16 GW (at ECT start) No diagnosis	7 ECTs	No data	Abdominal pain after 3rd ECT and after last ECT	Baby born full term, normal	Contains summary of previous reports by others of ECT given under pregnancy, unclearly presented. Only one new case by the authors presented in table. Incomplete reference list, impossible to trace many references
Evrard (1961)	Case Netherlands	27 years, P2, GW 31–35 (8 months pregnant) Manic depressive psychosis Previous history of depression	6 ECTs over 3 weeks and discharged	No data	Normal delivery	Baby boy born full term, normal, healthy followed for 6 years	Post partum relapse, readmitted and given 12 ECTs with antipsychotic medication (Tofranil), improved and discharged
Barten (1961)	Cases *N* = 2 Netherlands	Case 1: 36 years, P4, GW 32–36 Endogenous depression with psychotic features Case 2: 33 years, P2, GW 31–34 Obsessive compulsive disorder	Case 1: 10 ECTs Case 2: 8 ECTs	Case 1: Anesthesia: Pentothal and muscle relaxant (succinylcholine chloride). FHR monitoring, frequency changes during ECT Case 2: Anesthesia type unknown, succinylcholine noted. FHR monitoring	Case 1: In 7–8 ECT, at 34 GW, uterus also in constant contraction. On 10th shock no uterine contraction. Spontaneous delivery 5 weeks after last ECT and 1 week after due date Meconium-stained amniotic fluid. Case 2: FHR deceleration. Patient had slight visible cyanosis lasting 30 s after ECT. Patient went into labor 12 days before date	Case 1: Baby boy, 3,450 g healthy. Some degree of fetal oxygen deficiency during shocks due to FHR changes and meconium-stained amniotic fluid Case 2: Baby girl, 3,000 g “normal impression.” Amniotic fluid clear	Case 1: 6 weeks after birth patient in reasonably good psychological state, discharged
Ferrari (1960)	Cases *N* = 8 Italy	Case 1: 19 years, P1, GW 18 (5 months) Depression, delusions of guilt (condition several years prior, symptom worsening during pregnancy) Case 2: 28 years, P3, GW 31 (8 months) Unstable mood (about 2 years prior to pregnancy) Case 3: 32 years, P2, GW 18 (5 months) Severe depression (after sudden unexpected neonatal child loss 5 days old, in 1st pregnancy 1 year prior) Case 4: 22 years, P2, GW 22 (6 months) Severe depression Case 5: 21 years, P1, GW 18 (5 months) Major depression (with suicide attempts) Case 6: 35 years, P2, GW 22 (6 months) Severe depression (Accidental contact pregnancy) Case 7: 25 years, P2, GW 9 (3 months) Severe depression, anxious meloncholia (Spontaneous abortion in 1st pregnancy) Case 8: 27 years, P2, GW 31 (8 months) Severe depression (prior to symptoms, death of 6 year old son during current pregnancy)	Case 1: 7 ECTs (3 times weekly) Case 2: 9 ECTs Case 3: 10 ECTs Case 4: 9 ECTs Case 5: 7 + 3 ECTs Case 6: 10 ECTs Case 7: 2 + 6 ECTs Case 8: 7 ECTs	No data	Case 1: modest improvement. Normal pregnancy and birth at 8½ months Case 2: improvement, delivery 10 days after last ECT treatment Case 3: moderate improvement. Delivery at 8½ months. Postpartum symptom recovery. Case 7: Vaginal bleeding after 2 ECTs. After 15 day pause, another 6 ECTs given. Case 8: 3 days after last ECT spontaneous birth	7 baby children reported ok – no abnormalities. Case 8: baby in good condition Case 7: 1 Neonatal death at 8 days due to bronchopneumonia	All case data sparse, with modest symptom improvement Case 1: 20 days postpartum relapse of symptoms and another 8 ECTs. Case 7: postpartum treated with additional 10 ECTs Recommends ECT in pregnancy
Sobel (1960)	Cases *N* = 33 USA	No age data except for 2 infant deaths, to mothers a) 42 years and b) 37 years ECT indication: States of severe agitation and/or catatonia. ECT administered as an emergency form of treatment Retrospective hospital chart study of ECT treated patients while pregnant who delivered in 8 New York state hospitals from 1949 to 1958	No data on type or amount of ECT given to each case. No pregnancy term or GW data, except for 2 cases with post ECT abdominal pain in 31–35 GW (8 months pregnancy)	2 cases of severe recurrent abdominal pain directly following ECT in 31–35 GW One breech presentation delivery	Spontaneous or induced abortions, reported as none	31 Babies. All with birth weight over 2,500 g (no premature babies). Fetal damage among ECT treated is reported as 6 % - but type of damage not specified. 2 infant deaths: 1 anencephali (born to mother a); 1 congenital cysts and bronchopneumonia (born to mother b and one of twins)	Overall sparse data and unclear. Fetal abnormality 6 % is commented as surprisingly high – and data otherwise lacking. Follow-up on babies from 2 weeks to 5 months reported having no abnormalities
Schachter (1960)	Case France	34 years, GW 8 (2nd month pregnant) Depression	24 ECTs	No data	No data	Baby girl 2,000 g, premature, cyanotic in need of resuscitation, 34 GW. Severe mental retardation, congenital glaucoma, left-sided cleft palate	Mainly case report about child seen at 4 to 7 years old. Some, but sparse data about mother
Smith (1956)	Cases *N* = 15 UK	Age range: 18–35 years Age mean: 27 years Case 1: P1, GW 16 Case 2: P1, GW 30 Case 3: P2, GW 28 Case 4: P2, GW 12 Case 5: P2, GW 8 Case 6: P1, GW 16 Case 7: P3, GW 30 Case 8: P3, GW 20 Case 9: P4, GW 20 Case 10: P3, GW 40 Case 11: P1, GW 30 Case 12: P1, GW 24 Case 13: P1, GW 33 Case 14: P6, GW 16 Case 15: P1, GW 4 Case 7: two previous miscarriages Case 9 Rhesus negative Diagnoses: 12 endogenous depression, 1 acute schizophrenic reaction, 1 paranoid schizophrenic syndrome	Case 1: 6 ECTs Case 2: 6 ECTs Case 3: 7 (m)ECTs Case 4: 6 ECTs Case 5: 6 ECTs Case 6: 5 (m)ECTs Case 7: 4 ECTs Case 8: 5 (m)ECTs Case 9: 4 (m)ECTs Case 10: 5 ECTs Case 11: 6 (m)ECTs Case 12: 5 (m)ECTs Case 13: 5 ECTs Case 14: 6 ECTs Case 15: 6 ECTs (m) = modified ECT	Anesthesia, i.e. modified (m)ECT, given in 5 cases, all with thiopentone and muscle relaxant suxemethonium All 7 other cases unmodified ECT, i.e., without anesthesia	No induced labour and miscarriages reported as none, except uncertainty for case 7 and in case 2 prolonged labor	All children followed up between 11 months 5 years. Two children with neurotic traits. Intellectual deficiencies and physical abnormalities reported as none	Case 9 (Rhesus negative) no report of any complications
Monod (1955)	Cases *N* = 4 France	Case 1: 28 years, P2, GW 20 Depression. Also treated with Largactil medication Case 2: 34 years, P1, GW 12 Depression Case 3: 19 years, P1, GW 20 Confusion state Case 4: 25 years, P1, GW 4 Confusion state	Case 1: 2 ECTs Case 2: 4 ECTs Case 3: 3 ECTs Case 4: 9 ECTs ECT frequency 1× weekly	Case 4: Pentothal anesthesia and curare. Improvement of symptoms after 3rd ECT. A long apnea after 6th ECT	Case 1: Normal term delivery Case 2: Delivery with aid of forceps due to changes in heart sound Case 3: Normal birth Case 4: No data	Case 1: Birth of daughter. Case 2: Baby boy, 3,250 g. At 9 months old healthy Case 3: Healthy baby boy Case 4: No baby data	Case 2: Postpartum symptom relapse requiring treatment
Laird (1955)	Cases and review *N* = 8 USA	Case 1: 24 years, P3, GW 8–39 Hebephrenic schizophrenia Case 2: 37 years, P1, GW20-28 Psychotic depression Case 3: 39 years, P2, GW 0–8 Schizoaffective Case 4: 29 years, P1, GW 20–40 Schizoaffective Case 5: 35 years, P4, GW 38. Manic-depressive disorder, depressed Case 6: 28 years, P3, GW 16–24 Paranoid schizophrenia Case 7: 19 years, P1, GW 26–34. Catatonic schizophrenia Case 8: 20 years, P1, GW 16–28 Schizoaffective	Case 1: 18 ECTs Case 2: 28 ECTs between 18–30 GW +7 ECTs after GW31 Case 3: 7 ECTs Case 4: 17 ECTs Case 5: 4 ECTs Case 6: 20 ECTs Case 7: 7 ECTs Case 8: 25 ECTs	All unmodified ECT (without anesthesia)	Case 1: Delivery 1 month after last ECT Case 2: Delivery 2 days after last ECT at GW 34 Case 4: Delivery 7 days after last ECT Case 5: Last ECT 2 weeks before delivery Case 6: Delivery 4 months after last ECT Case 7: Caesarian section due to platypelloid pelvic and left shoulder presentation, at 8½ months (36 GW), 14 days after last ECT Case 8: Delivery 2 months after last ECT	Case 1: Full term baby, (no weight) Case 2: Baby girl, preterm (GW34), 2,100 g, normal development Case 3: Full term baby, 3,000 g Case 4: Full term baby, 3,500 g Case 5: Full term baby, 2,900 g Case 6: Full term baby, 3,700 g Case 7: Baby girl, 3,400 g Case 8: Full term baby, (no weight)	Case 1: Pregnancy suspected but examination impossible in first 2 months due to mental condition ECT during pregnancy viewed as safe
Russell and Page (1955)	Cases *N* = 10 UK	14-35 GW (3 to 8½ months pregnant)	ECT given between 14–35 GW (3 to 8½ months)	No data	No data	No data	Commentary, letter to editor with very sparse data. No adverse effects reported
Charatan and Oldham (1954)	Case (and review of 12 cases) UK	29 years, GW 16 (at admission) GW 28 (at ECT start)—31GW Catatonic schizophrenia	6 ECTs (between 28–31 GW) 2 times weekly Device: Strauss-McPhail (Theratronics Ltd.)	Anesthesia: Pentothal and suxethonium	Labor uneventful	Baby full term, 3,500 g	Mental state temporarily improved
Wickes (1954)	Case UK	No age, P8, approx 20 GW when ECT treated Schizophrenia	2 ECTs 35 insulin comas in 1st and 2nd trimester. ECTs given 1 month after insulin coma	No data	No data	Baby born 4 weeks before estimated term Child examined at 3 years, severe mental deficiency, blind in left eye, unable to feed himself, talk or stand, incontinent	Only 2 ECTs, mainly insulin coma treatment. Fetus exposed to insulin coma treatment in first trimester, pregnancy unknown until third trimester
Yamamoto et al. (1953)	Case USA	25 years, P2, GW 18–21 (5 months pregnant) Schizophrenic reaction First born child died 1 year earlier	12 ECTs Dismissed from hospital 2 months after last ECT	No data	Labor and delivery normal, 3 weeks after left hospital	Baby girl examined at 32 months. Child slow in sitting up, walking late (15–18 months), verbally one word syllables, temper fits, active, chewing fingernails, sleeping difficulties, little interest in pictures and other children, eye strabismus, and concluded mentally retarded	Patients progress after ECT described satisfactory and clear mentally
Forman et al. (1952)	Cases *N* = 2 USA	Case 1: 22 years, P2, GW 20 Depression (Retrograde amnesia accident depression) Case 2: 43 years, P1, GW 24–32 Reactive depression	Case 1: 7 ECT Case 2: 9 ECTs 8 major convulsions, 3 petit mal	No data	Case 1: Delivery at full –term without depression Case 2: Great improvement, then worse again. At 38 GW caesarian section. Phlibitus deep vein thrombosis in left leg	Case 1: Baby, 6 lb 2 oz Case 2: Baby, 5 lb 4 oz	Case 2: Several postpartum ECTs
Cooper (1952)	Case South Africa	28 years Psychotic depression (suicidal event, auditory hallucinations) (case admitted in 1951)	9 ECTs administered in 3rd semester 3 times weekly		9 h after last ECT normal labor occurred	Baby 7 lb Healthy infant	Mental status not improved
Porot (1949)	Cases *N* = 3 Alger	Case 1: ECT given early in pregnancy. Retarded condition. Case 2: GW 28 (7 months pregnant) Agitated condition. Case 3: Melancholic state	Case 1: 10 ECTs Case 2: 3 ECTs Case 3: 12 ECTs and 23 insulin-comas	Case 2: Vaginal bleeding after 3rd ECT. Phlebitis in patients leg, ECT discontinued Case 3: Vaginal bleeding after 1st ECT	Case 2: Normal delivery	Case 1: Baby full term Case 2: Baby healthy Case 3: Baby full term	Sparse data. Author refers to another known case given 7 ECTs during 3rd pregnancy month, terminating in an abortion
Plenter (1948)	Cases *N* = 3 Netherlands	Case 1: 32 years, P5, GW 8 (at admission) GW 14 (at ECT start). Schizophrenia, melancholic syndrome (Psychotic with strong anxiety) Last 4th child born recently. Case 2: 32 years, GW 10 (at admission) GW 14 (at ECT start). Mania, psychotic Case 3: 26 years, P1, GW 24–38 Psychosis, suicidal	Case 1: 6 ECTs in 2nd trimester (+ 7 ECTs after miscarriage) Case 2: 18 ECTs in 2nd trimester Case 3: 23 ECTs (2 times weekly)	Case 1: Strong vaginal bleeding and miscarriage in the night after 6th ECT. Placenta had to be removed manually Case 2: Normal delivery Case 3: Abdominal, belly pain after 1st ECT		Case 2: Baby boy, born full term. Case 3: Baby girl	Case 1: Worsening of symptoms after miscarriage, given further 7 ECTs and then dismissed
Simon (1948)	Cases *N* = 3 USA	Case 1: 36 years, 14–17 GW Agitated depression Case 2: 25 years, 18–34 GW Anxiety attacks Case 3: 25 years, GW 22–26 (6th months pregnant) Agitated depression with somatic delusions	Case 1: 6 ECTs, 5 grand mal seizures (at time of first ECT almost 4th month pregnant) Case 2: 10 ECTs between 18–34 GW and 4 ECTs later due to relapse. Case 3: 11 ECTs (altogether 13 convulsions, including insulin therapy)	No data	Case 1: Pregnancy described “stormy and toxic”. Last ECT given 7 months before delivery Case 2: Delivery 10 days after last ECT Case 3: Delivery 29 days after last ECT	Case 1: Child died 2 days after birth, cause unknown Case 2: Baby boy described consistently healthy Case 3: Baby girl healthy	Case 1: Not seen again after 5 months pregnant but replied to questionnaire 1 year and 5 months later. Case 2: Further 12 ECTs post partum and improved Case 3: Given Sub-shock insulin treatment early in pregnancy
Doan and Huston (1948)	Cases *N* = 7 USA	Case 1: 32 years, P5, GW 12–16 (2 months pregnant) Depression Case 2: 35 years, P7, GW 16 Recurrent depression Case 3: 27 years, P4, GW 28 Psychotic. Blood and spinal fluid examination with Wassermanns test positive (possible infection/syphilis). Given penicillin treatment without improvement, thereafter ECT Case 4: 24 years, P2, GW 24 (6th month pregnant) Psychosis Case 5: 31 years, P2, GW 12 Delusional Case 6: 24 years, P1, GW 27 Psychosis Case 7: 40 years, P5, GW 27 Psychosis	Case 1: 6 ECTs Case 2: 10 ECTs Case 3: 2 ECTs Case 4: 9 ECTs Case 5: 18 ECTs Case 6: 12 ECTs Case 7: 16 ECTs ECT frequency 2–3 times weekly	No anesthetic agent, but muscle relaxant curare given before each treatment. ECT voltage set at 120 and 60-cycle current (sine wave) applied for 0.1-0.2 s. Each treatment produced a major convulsion	Case 1: Normal delivery at 36 GW Case 2: Normal delivery at 36 GW Case 3: Delivery normal Case 7: Labor induced at 36 GW, normal delivery	Case 1: Baby ok. Case 2: Baby examined 2 months later, development reported normal Case 3: Normal infant Case 4: Normal infant, follow-up at 18mnths, no developmental abnormalities Case 5: Normal infant, 9 months later followed up, doing well Case 6: Normal child Case 7: Normal infant, followed up at 7 months, baby reported normal	Case 1: mother improved Case 2: mother improved Case 3: Antiluetic (anti-syphilis) treatment after delivery Case 4: ECT gave no symptom improvement Case 5: moderate symptom improvement from ECT, at 9 months postpartum still mentally ill. Case 6: symptoms improved after ECT, but at 8 months postpartum still mentally ill. Case 7: very slight symptom improvement from ECT
Boyd and Brown (1948)	Cases *N* = 2 USA	Case 1: 17 years, P2, GW 17–18 (4½ months pregnant) Schizophrenia with hebephrenic and catatonic, features. Case 2: 20 years, P1, GW 27–30 (7 months pregnant) Manic-depressive psychosis (bipolar)	Case 1: 26 ECTs with curare medication Case 2: 2 ECTs without curare and grand-mal induced seizure	Case 1: After 2nd ECT vaginal bleeding. No vaginal bleeding after 3rd ECT. Case 2: After 1st ECT, tonic contraction of uterus, lasting 10 min and vaginal bleeding. After 2nd ECT vaginal bleeding with blood clots and sustained uterus contraction 15 min	Case 1: Obstetric examination normal progress of pregnancy. No delivery data. Case 2: FHR increase during 2nd ECT, inaudible. Premature labor 4 days after 2nd ECT	Case 1: No child data. Case 2: Baby boy 5¼ lb, premature and nothing unusual noted	Case 1: ECT failed to give complete recovery. Case 2: 14 more ECTs given in postpartum period due to relapse of symptoms. Recovery made and thereafter discharged
Block (1948)	Case USA	30 years, P1, 18–21 GW (ECT start when 5 months pregnant) Depressed, psychotic	26 ECTs, started at 3 times weekly first 2 weeks, then 2 times weekly. Recovered for a period of 2 months then relapsed, ECT treatment resumed until 6 days before delivery	No data	No data	Baby born, no other data	4 ECTs in postpartum period (Given a total amount of 30 ECTs)
Kent (1947)	Cases *N* = 3 New York, USA	Case 1: 35 years, P4, GW unknown. Dementia praecox, paranoid type Case 2: 31 years, GW 18–21 (5 months pregnant) at admission and GW 22–26 (6 months pregnant) at ECT start. Manic- depressive psychosis, manic type Case 3: 33 years, P4, GW 14–17 GW (4 months pregnant). Dementia praecox, paranoid type	Case 1 : 16 ECTs and 50 days of insulin coma treatment ECT 3 times weekly and daily insulin-coma Case 2: 30 ECTs, 3 times weekly (26 grand-mal and 4 petit mal seizures). Case 3: 20 ECTs, 3 times weekly, insulin-coma at GW 14–17, and 90 insulin-coma treatments with 80 comas	No data	Case 1: No info Case 2: Caesarian section at 8½ months pregnancy Case 3: Spontaneous labor, vaginal delivery 2 months after ended ECT and coma treatment	Case 1 : Miscarriage (abortion), fetus 6 in. Case 2: Normal child, 6 lb (3,000 g) Case 3: Baby 7½ lb	Case 1: Treatment suspended for 10 days after abortion. Case 2: 7 ECTs postpartum
Gralnick (1946)	Case (1 ECT and 1 insulin coma case) USA	Case 1: 31 years, P5, GW 1–13 Catatonic, mute refusing to eat. History of previous 19 insulin shock treatments. [Case 2, insulin shock: 32 years, P5. Auditory hallucinations, 6 weeks after admission pregnancy confirmed. History of personality changes past 6 years]	Case 1: 6+ ECTs (unclear pregnancy length, ECT given in 1st trimester) Also 18 insulin treatments with 8 comas	No data	Case 1: In 3rd trimester, delivery noted as spontaneous of macerated fetus	Case 1: Macerated fetus weight 7 lb 10 oz. (delivered in 3rd trimester)	Report of 2 cases, but only 1 with ECT and insulin coma [Case 2: 25 Insulin coma treatments, begun in 1st trimester—14 moderate deep comas (30–60 min), hypoglycemic periods (4–5 h) with Fetus death.]
Polatin and Hoch (1945)	Cases *N* = 2 USA	Case 1: 28 years, P2, GW 15 Manic depressive disorder, depressed (Uncooperative for psychotherapy treatment before ECT) Case 2: 27 years, P(unknown), GW 29 (at ECT start), GW 20 (at admission) Psychoneurosis, conversion hysteria with depression. Psychotherapy treatment before ECT	Case 1: 6 ECTs (5 convulsions) Case 2: 10 ECTs (started at 7 months pregnant)	No data	Case 1: Spontaneous delivery after 9 h of labor. Case 2: Spontaneous delivery after 21 h of labor No miscarriages, no premature labor, no evidence of asphyxia of children	Case 1: Baby boy, 3,270 g. No abnormalities detected. Baby progress normal. Case 2: Normal boy infant, 3,470 g. No abnormalities detected. Baby progress normal	
Thorpe (1942)	Case UK	23 years, P3 (2nd pregnancy spontaneous abortion) 17–18 GW at admission Acute agitated melancholia	13 ECTs given over 6 weeks, treatment started 5 weeks after admission (at approx. 23 GW)	No data	No delivery data	No baby data except patient discharged with a healthy 7 months old baby	

## Abbreviations

BL: Bilateral
BH: Bjørg Høie
BPM: Beats (heart beats) per minute
DSM-IV: Diagnostic Statistical Manual of Mental Disorders, fourth edition
ECT: Electroconvulsive therapy
EEG: Electroencephalogram
FHR: Fetal heart rate
GW: Gestation weeks
ICD-10: International Classification of Diseases, 10th revision
IH: Ingrid Harboe
KAL: Kari Ann Leiknes
KTH: Karianne Thune Hammerstrøm
LJS: Lindy Jarosch-von Schweder
M: Mean
MJC: Mary Jennifer Cooke
MRI: Magnetic resonant imaging
OCD: Obsessive Compulsive Disorder
SD: Standard deviation
UL: Unilateral
WWE: Women with epilepsy
